# Combination of the natural compound Periplocin and TRAIL induce esophageal squamous cell carcinoma apoptosis in vitro and in vivo: Implication in anticancer therapy

**DOI:** 10.1186/s13046-019-1498-z

**Published:** 2019-12-21

**Authors:** Lujuan Han, Suli Dai, Zhirong Li, Cong Zhang, Sisi Wei, Ruinian Zhao, Hongtao Zhang, Lianmei Zhao, Baoen Shan

**Affiliations:** 1 Research Centre, the Fourth Hospital of Hebei Medical University, 12# Jiankang Road, Shijiazhuang, 050011 Hebei China; 2 Department of Pathology and Laboratory Medicine, Perelman School of Medicine, University of Pennsylvania, Philadelphia, PA 19104 USA

**Keywords:** TRAIL, CPP, ESCC, DRs, Wnt/β-catenin pathway

## Abstract

**Background:**

Esophageal cancer is one of the most common malignant tumors in the world. With currently available therapies, only 20% ~ 30% patients can survive this disease for more than 5 years. TRAIL, a natural ligand for death receptors that can induce the apoptosis of cancer cells, has been explored as a therapeutic agent for cancers, but it has been reported that many cancer cells are resistant to TRAIL, limiting the potential clinical use of TRAIL as a cancer therapy. Meanwhile, Periplocin (CPP), a natural compound from dry root of *Periploca sepium* Bge, has been studied for its anti-cancer activity in a variety of cancers. It is not clear whether CPP and TRAIL can have activity on esophageal squamous cell carcinoma (ESCC) cells, or whether the combination of these two agents can have synergistic activity.

**Methods:**

We used MTS assay, flow cytometry and TUNEL assay to detect the effects of CPP alone or in combination with TRAIL on ESCC cells. The mechanism of CPP enhances the activity of TRAIL was analyzed by western blot, dual luciferase reporter gene assay and chromatin immunoprecipitation (ChIP) assay. The anti-tumor effects and the potential toxic side effects of CPP alone or in combination with TRAIL were also evaluated in vivo.

**Results:**

In our studies, we found that CPP alone or in combination with TRAIL could inhibit the proliferation of ESCC cells and induce apoptosis, and we certificated that combination of two agents exert synergized functions. For the first time, we identified FoxP3 as a key transcriptional repressor for both DR4 and DR5. By down-regulating FoxP3, CPP increases the expression of DR4/DR5 and renders ESCC cells much more sensitive to TRAIL. We also showed that CPP reduced the expression of Survivin by inhibiting the activity of Wnt/β-catenin pathway. All these contributed to synergistic activity of CPP and TRAIL on ESCC cells in vitro and in vivo.

**Conclusion:**

Our data suggest that CPP and TRAIL could be further explored as potential therapeutic approach for esophageal cancer.

## Background

Esophageal cancer is one of the most common malignant tumors in the world, accounting for 508,585 deaths worldwide in 2018 [1]. Esophageal cancer can be categorized into esophageal squamous cell carcinoma (ESCC) and adenocarcinoma (EAC). ESCC is the major histological type of esophageal cancer in Asia [2], and studies have shown that about 59–93% of ESCC patients have a TP53 mutation in genomic [3] and 23% of ESCC patients shared deregulation of Wnt/β-catenin activity [4]. More than half of patients have distant metastases when they are diagnosed [5]. Even with current therapies, the prognosis of esophageal cancer is not favorable, with overall 5-year survival rate ranging from 20 to 30% [6]. A number of genes with abnormal expression in ESCC have been identified, including TP53 and EGFR. Though TP53 mutation occur at a high rate and could be a potential target for ESCC, targeting TP53 mutation has been unsuccessful so far. Therefore, other potential therapeutic targets of ESCC need to be further explored [7, 8].

The TNF-related apoptosis-inducing ligand (TRAIL), also called TNFSF10 and APO2L, is a member of the tumor necrosis factor superfamily (TNFSF). The human TRAIL is the only TNFSF member that binds to four membrane receptors and one soluble receptor [9]. The receptors for human TRAIL can be divided into two classes. One widely expressed class of receptors contains a full-length intracellular death domain that induces apoptosis, called death receptors (DRs), including TRAIL-R1 (also known as DR4 and TNFRSF10A) [10] and TRAIL-R2 (also known as DR5 and TNFRSF10B) [11–17]. Another class contains the alternative receptors TRAIL-R3 (also known as DCR1 and TNFRSF10C) [16–19], TRAIL-R4 (also known as DCR2 and TNFRSF10D) [20, 21] and osteoprotegerin (OPG, also known as TNFRSF11B) [22].

TRAIL is capable of inducing apoptosis of cancer cells without causing much adverse effects in xenografted tumor models, showing as a promising anti-tumor strategy [9, 23–25]. Despite the promising in vivo experiment data, TRAIL fails to demonstrate satisfactory activities in clinical trials. One of vital reasons is that many cancer cells unfortunately acquire resistance to TRAIL-induced apoptosis [26]. To date, some mechanisms by which cancer cells developed resistance to TRAIL have been elucidated, including the dysfunction of DR4 or DR5, defects in FADD and the overexpression of anti-apoptotic proteins such as c-FLIP, Bcl-2 and Survivin [27]. Therefore, it is urgent to find ways to enhance the sensitivity of cancer cells to TRAIL.

Periplocin (CPP) is isolated from the extract of cortex periplocae (CPP), which is the dry root of *Periploca sepium* Bge. As a traditional herbal medicine, CPP’s cardiotonic and diuretic activity have been well recognized [28]. Recent studies have shown that CPP can inhibit the proliferation and promote the apoptosis in several cancer cells [29–31]. Previously we have shown that CPP has the anti-tumor activity in gastric cancer and colon cancer [29, 31], partly through inhibition of the Wnt/β-catenin pathway [29, 32]. In other studies, CPP was found to induce the expression of DRs and enhance TRAIL-induced apoptosis in hepatocellular carcinoma cells that were resistant to TRAIL, while the mechanism of which is still unclear until now [33].

In this study, we are committed to finding the mechanism of drug resistance of TRAIL in ESCC and explore the effective drug combination for the treatment of ESCC. Our data showed that most of ESCC cells we tested are resistant to TRAIL, but sensitive to CPP. A synergistic anti-proliferation activity and anti-tumor activity was observed when ESCC cells or xenografted tumors were treated by TRAIL and CPP. Notably, we firstly identified FoxP3 as one of the important transcription factor of DRs in the ESCC, and revealed that suppression of FoxP3 expression is the essential molecular mechanisms for CPP to increase DRs Expression. Therefore, our study reveal a new mechanism of TRAIL resistance in ESCC and point to an effective therapeutic strategy for ESCC: a combination of TRAL and CPP.

## Materials and methods

### Cell lines and culture

ESCC cell lines Eca-109 and TE-1 were obtained from the Shanghai Institute for Biological Sciences. YES-2, KYSE-30, KYSE-410 and KYSE-510 cell lines were kindly provided by Professor Masatoshi Tagawa (Department of Molecular Biology and Cancer Biology, Chiba University, Japan). KYSE-150, KYSE-180 and KYSE-450 cell lines were gifts from the Zhan Qimin’s lab from the Cancer Hospital of Chinese Academy of Medical Sciences (Beijing, China). All cells were cultured in RPMI 1640 medium (GIBCO, USA) supplemented with 10% heat-inactivated fetal bovine serum (Biological Industries, Beit-Haemek, Israel), penicillin and streptomycin (Invitrogen) and incubated at 37 °C in air containing 5% CO_2_.

### Chemicals and reagents

Periplocin (purity ≥96%) was obtained from the New Drug Research and Development Center of North China Pharmaceutical Group Corporation (Hebei, China). Periplocin was dissolved in DMSO and diluted to 50, 100 and 200 ng/ml using RPMI-1640 medium (final concentration of DMSO < 0.01%). Tumor necrosis factor-related apoptosis-inducing ligand (TRAIL) was obtained from QiaEr (Shanghai, China).

### RNA isolation and quantitative real-time PCR (qRT-PCR)

Total RNA was extracted from cells with TRIzol Reagent (Invitrogen) following manufacturer’s instructions. cDNA was synthesized from total RNA using GoScript™ Reverse Transcription System (Promega, USA) according to the manufacturer’s protocol. Quantitative real-time PCR was conducted using a SYBR Green PCR Kit (Promega, USA) with a real-time PCR system (ABI 7500). PCR-based amplification was performed using the primers listed in Additional file 2: Table S1. RNA expression levels were normalized to GAPDH expression levels. Relative expression levels were calculated using the 2^-ΔΔCt^ method.

### Cell proliferation assay

The MTS assay was performed according to the manufacturer’s instructions to examine the proliferation of eight esophageal cancer cell lines. Cells (1 × 10^4^ cells/well) were plated in 96-well plates with 6 replicates/group. Cells were incubated for 24 and 48 h in the presence of different concentrations of drugs. At each of the desired time points, MTS solution (Promega, USA) was added (20 μl/well) into each well and incubated for 2 h at 37 °C in the dark, followed by measurement of the absorbance at 492 nm with a microplate reader. We selected 8 ESCC cell lines: Eca-109, YES-2, TE-1, KYSE-30, KYSE-150, KYSE-180, KYSE-410 and KYSE-510 cells to examine the synergistic activity of CPP and TRAIL. To calculate the combination index (Q value), we used the formula: Q value = C / (A + B – A × B), in which A represents the inhibition rate of drug 1, B is the inhibition rate of drug 2, and C was the total inhibition rate of combined drugs group.

### Flow cytometry assay

Treated and untreated YES-2, KYSE-150 and KYSE-510 ESCC cells were harvested in PBS. Then, the cells were incubated with Annexin V-PE and 7-AAD (BD Pharmingen™ PE Annexin V Apoptosis Detection Kit I) for 15 min at room temperature in a darkroom. The stained cells were then analyzed using flow cytometry (BD FACSCalibur, USA).

Flow cytometry was also used to determine changes in the expression of DR4 and DR5 at the cell membrane after treatment with various concentrations of CPP. The treated and untreated cells were stained with phycoerythrin-conjugated mouse monoclonal anti-human DR4 (eBioscience, CA, USA) or DR5 (eBioscience, CA, USA) antibodies for 30 min at 4 °C. The stained cells were analyzed using flow cytometry (BD FACS Calibur, USA).

### TUNEL assay

The treated YES-2, KYSE-150 and KYSE-510 ESCC cells were harvested in PBS. Cell apoptosis was detected by TUNEL staining using a kit (In Situ Cell Death Detection Kit, Fluorescein, Cat. No. 11684795910, Roche, Germany) according to the manufacturer’s protocol. After washed with PBS, samples were subjected to immunofluorescence microscopy.

### Immunohistochemistry

Tumor sections (5 μm) were dewaxed in xylene and hydrated through a graded ethanol series. For antigen retrieval, slides were boiled in EDTA (1 mM, pH 9.0) for 10 min in a microwave oven. Endogenous peroxidase activity was quenched with 0.3% hydrogen peroxide solution for 10 min at room temperature. After rinsing with PBS, slides were blocked with 5% BSA for 30 min. Slides were subsequently incubated with polyclonal antibodies against DR4, DR5 (CST, USA), FoxP3 (Abcam, USA), and β-catenin (CST, USA) overnight at 4 °C. The next day, sections were incubated with the secondary antibody for 30 min at 37 °C followed by incubation with an HRP-labeled streptavidin solution for 30 min. PBS was used to wash the samples after each step. After visualization of the positive antigen antibody reaction by incubation with 3, 3-diaminobenzidine-tetrachloride (DAB) for 5 min, sections were counterstained with haematoxylin and evaluated by light microscopy.

### Western blot analysis

Western blot analysis was carried out using the standard method. Cells were homogenized and centrifuged. Proteins were separated by 10% SDS-PAGE, and transferred electrophoretically onto PVDF membranes (Millipore, Billerica, MA, USA) for incubation with antibodies against DR4, DR5, c-FLIP, XIAP, FADD, survivin, β-catenin, p-β-catenin (Ser675) (CST, USA), Bcl-2, Bax (Proteintech, China), cleaved Caspase-3 (BioWorld, USA), FoxP3 (Abcam, UK). The membranes were incubated with the fluorochrome-labeled secondary anti-rabbit IgG (IRDye 800-LI-COR, Odyssey) for 1 h at room temperature. The membranes were visualized using the Odyssey Infrared Imaging System (LI-COR, USA). GAPDH was used as a loading control. Western blotting analysis was independently repeated 3 times.

### Plasmid transfection

Cells were seeded in 6-well plates (2 × 10^5^ cells/well) or 100-mm dishes (2 × 10^6^ cells/dish) one day before transfection. Transfection was performed when cells were at 60–80% confluence. Cells were transfected with the plasmids as listed in each figure. All transfections were performed using Lipofectamine 2000 (Invitrogen, Carlsbad, CA, USA) following the manufacturer’s instructions. To minimize toxicity, the transfection complex medium was replaced with fresh medium after 6 h. Analyses of the recipient cells or further experiments were performed 48 h after transfection.

### ChIP assay

For the ChIP assay, 2 × 10^6^ treated and untreated YES-2 and KYSE-150 ESCC cells were prepared with a ChIP assay kit (Millipore, Cat #17–10,086, Germany) according to the manufacturer’s instructions. The resulting precipitated DNA samples were analyzed by agarose gel electrophoresis after PCR.

### Dual-luciferase reporter assay

HEK293T cells were obtained from the Shanghai Institute for Biological Sciences and were cultured in DMEM (GIBCO, USA). Cells were plated in 24-well plates, co-transfected with FoxP3 plasmids, containing FoxP3 binding region plasmids or non-containing FoxP3 binding region plasmids and Renilla plasmids and treated with CPP (50, 100, 200 ng/ml). Cells were collected when treated with drugs 24 h after transfection 24 h, and luciferase activities were analyzed by the dual-luciferase reporter assay kit (Promega, Dual-Luciferase Reporter Assay System, E1910, USA). Reporter activities were normalized to the activity of the Renilla control.

Cells were plated in 24-well plates, co-transfected with TOP/FOP FLASH plasmids (MiaoLingBio, China) and Renilla plasmids and treated with LiCl (20 mM) (Sigma, USA) to activate the Wnt/β-catenin pathway. Cells were collected when treated with drugs 24 h after transfection 24 h, and luciferase activities were analyzed by the dual-luciferase reporter assay kit (Promega, Dual-Luciferase Reporter Assay System, E1910, USA). Reporter activities were normalized to the activity of the Renilla control.

### Nuclear and cytoplasmic protein extraction

For the extraction of nuclear and cytoplasmic proteins, cells were treated with NE-PER™ Nuclear and Cytoplasmic Extraction Reagents (Thermo Fisher Scientific, 78,833, USA) according to the manufacturer’s instructions.

### AAV-TRAIL

Recombinant adeno-associated virus vector for soluble TRAIL (AAV-TRAIL) is kindly provided by Professor Shi Juan from the Institute of Basic Medical Sciences, Chinese Academy of Medical Sciences & Peking Union Medical College. The less pathogenic Adeno-associated virus (AAV)-based vectors are efficient for gene deliver. Therefore, AAV-TRAIL is used to increase the expression of TRAIL in vivo in mouse xenograft models. The process of constructing AAV-TRAIL is as described in the articles [34, 35].

### Animal experiments

Male athymic Balb/c nude mice (4–6 weeks, 20–25 g) were purchased from Vital River Laboratory Animal Technology (Beijing, China). All animal experiments were approved by the Animal Care Committee of the Fourth Hospital of Hebei Medical University. Animals were housed at the Fourth Hospital of Hebei Medical University Experiment Animal Centre. Approximately 5 × 10^6^ KYSE-150 cells in 200 μl PBS were subcutaneously injected into the right flank of each mouse. Tumor volume was monitored using a caliper once every two days. Once the tumors reached 100 mm^3^ as calculated by volume = (length × width^2^)/2, the mice were randomly divided into six groups with five mice per group. The first group of mice were intraperitoneally injected with 0.9% physiological saline every two days, the second group of mice were intratumourally injected with 1 × 10^10^ Gps AAV-TRAIL, the third group of mice were intraperitoneally injected with 0.09 mg CPP every two days, and the fourth group of mice were intraperitoneally injected with 0.36 mg CPP every two days. After the intratumoural injection of 1 × 10^10^ Gps AAV-TRAIL into the fifth group of mice, 0.09 mg of CPP was intraperitoneally injected every two days, and the sixth group of mice were intraperitoneally injected with 0.04 mg cisplatin every two days. The tumor volumes were measured every two days. Mice were killed by spinal dislocation once they had been treated seven times. Then, the tumors were removed and weighed, and the tumors and organs (such as the heart, liver, spleen, lungs and kidneys) were fixed in formalin for immunohistochemical (IHC) analysis and morphological analysis.

### Statistical analysis

Statistics were calculated using Prism (GraphPad v5.0) and SPSS (IBM, v21.0). Error bars indicate the standard error of the mean (SEM). One-way ANOVA was used to examine the significant differences in the experimental data between the groups. *P* values were indicated in each figures: * *P* < 0.05, **P < 0.01, *** *P* < 0.001. P < 0.05 was regarded as significant. All experiments were independently repeated three times.

## Results

### CPP exhibited synergistic effect with TRAIL to reduce viability of ESCC cells

**Fig. 1:**
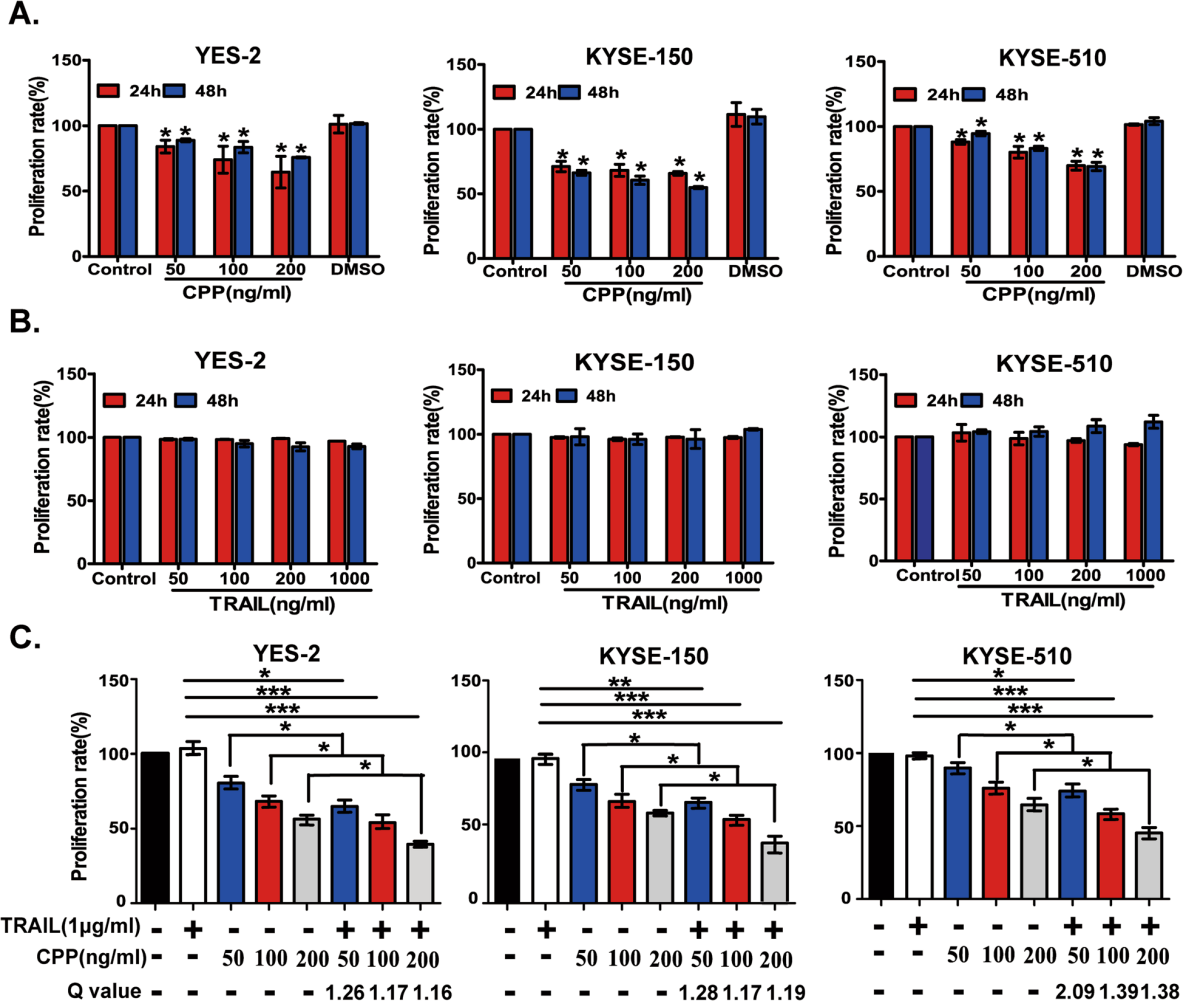
The effects of CPP and TRAIL alone or in combination on the viability of ESCC cells. **a.** ESCC cells (YES-2, KYSE-150 and KYSE-510) were treated with various concentrations of CPP and DMSO (< 0.01%) for 24 h or 48 h. Cell viability was determined by MTS assay. Data are shown as the mean ± SEM. *P < 0.05 compared to the untreated cell group. **b.** ESCC cells (YES-2, KYSE-150 and KYSE-510) were treated with various concentrations of TRAIL for 24 h and 48 h. Cell viability was determined by MTS assay. Data are shown as the mean ± SEM. **c.** ESCC cells (YES-2, KYSE-150 and KYSE-510) were treated with various concentrations of CPP alone or in combination with TRAIL for 24 h. Cell viability was detected by MTS assay. The Q values represents the combination index. Q values of more than 1.15 suggest a synergism in the combined treatment. Data are shown as the mean ± SEM. *P < 0.05, **P < 0.01, ***P < 0.001

To survey the activity of CPP on esophageal cancer cell lines, we firstly investigated the effect of CPP on the viability of 8 ESCC cell lines: Eca-109, YES-2, TE-1, KYSE-30, KYSE-150, KYSE-180, KYSE-410 and KYSE-510. Cells were treated with different concentrations of CPP (0, 50, 100 or 200 ng/ml) for 24 h and 48 h. Dose-dependent inhibition of cell viability was observed in all ESCC cell lines (Fig. 1a and Additional file 1: Figure S1A).

We next determined the sensitivity of TRAIL on these cell lines. Cells were treated with different concentrations of TRAIL (0, 50, 100, 200 or 1000 ng/ml) for 24 h and 48 h. In contrast to the sensitivity to CPP in all these cell lines, the viability of these cells did not change significantly even in the presence of 1000 ng/ml TRAIL (Fig. 1b and Additional file 1: Figure S1B), suggesting that most of ESCC cell lines were insensitive for TRAIL for unclear reasons.

We further selected 8 ESCC cell lines to examine the synergistic activity of CPP and TRAIL. Cells were treated with the combination of CPP (with various concentrations of 0, 50, 100 or 200 ng/ml) and TRAIL (1 μg/ml) for 24 h. The viability of the cells that received combined treatment was decreased significantly compared to treatment with CPP or TRAIL alone. The Q values (which represent the combination index) of all the combined treatments were more than 1.15, suggesting that there was synergism in the combined treatment of CPP and TRAIL. The Q values of YES-2, KYSE-150 and KYSE-510 cells were higher than that in other cell lines (Fig. 1c). In addition, these three cell lines were not sensitive to TRAIL (1 mg/ml), and the sensitivity to CPP was also lower than that of the other cell lines. Therefore, these three cell lines were selected for further study in subsequent experiments.

### CPP exerts synergistic activity with TRAIL to induce apoptosis in ESCC cells

**Fig. 2:**
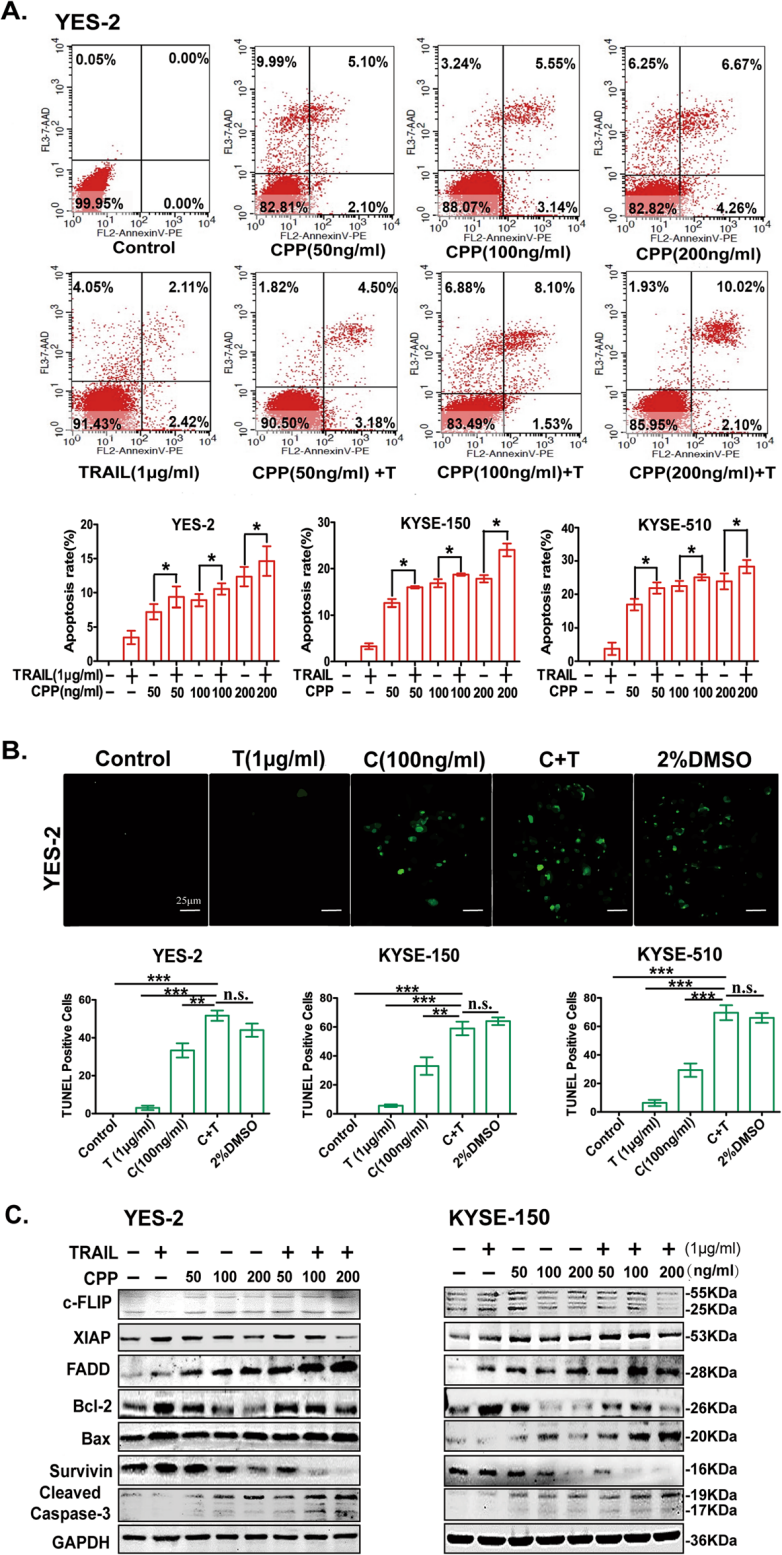
CPP and TRAIL induces apoptosis in ESCC cells. **a.** Flow cytometry analysis of YES-2, KYSE-150 and KYSE-510 cells treated with various concentrations of CPP alone or in combination with TRAIL for 24 h. The cells were examined by Annexin V-PE/7-AAD double staining. The lower right and the upper right quadrants indicate the percentage of apoptotic cells. Data are shown as the mean ± SEM. *P < 0.05. **b.** TUNEL staining of YES-2, KYSE-150 and KYSE-510 cells treated with CPP (100 ng/ml), TRAIL (1 μg/ml) and CPP combination with TRAIL for 24 h. The 2% DMSO treatment group served as a positive control. TUNEL-positive cells that show green fluorescence represent apoptotic cells. Scale bar, 25 μm. Data are shown as the mean ± SEM. **P < 0.01, ***P < 0.001. **c.** Western blot analysis of changes in the expression of apoptosis-related proteins in cells treated with CPP and TRAIL. YES-2 and KYSE-150 cells were treated with various concentrations of CPP alone or in combination with TRAIL for 24 h

We further examined whether CPP have synergistic activity with TRAIL to induce apoptosis. YES-2, KYSE-150 and KYSE-510 cells were treated with various concentrations of CPP (0, 50, 100 or 200 ng/ml), TRAIL (1 μg/ml), or the combination of CPP and TRAIL for 24 h, following which apoptosis rate was detected by flow cytometry. As shown in Fig. 2, the apoptotic rate of the cells that received combined treatment was significantly higher than that of the cells treated with CPP or TRAIL alone (Fig. 2a and Additional file 1: Figure S2A).

Similarly, the result of TUNEL assay showed that the number of TUNEL-positive staining of YES-2, KYSE-150 and KYSE-510 ESCC cells treated with CPP (100 ng/ml) and TRAIL (1 μg/ml) were all much higher than cells treated with CPP or TRAIL alone (Fig. 2b and Additional file 1: Figure S2B), which is consistent with the observed activity in proliferation assay.

TRAIL induces the activation of the death signaling pathway that involves many apoptosis-related proteins [26, 27]. To examine changes in these apoptosis-related proteins, YES-2 and KYSE-150 cells were treated with various concentrations of CPP (0, 50, 100 or 200 ng/ml), TRAIL (1 μg/ml) or the combination of both for 24 h, and cell lysates were collected for western blot analysis of c-FLIP, XIAP, FADD, Bcl-2, Bax, Survivin and Caspase-3. The results showed that FADD and cleaved Caspase-3 were upregulated dose-dependently after CPP treatment, while Survivin was significantly downregulated after CPP treatment. In addition, changes of proteins in the combination treatment group were much higher than that in CPP alone group (Fig. 2c). Changes of protein levels in these three proteins are consistent with the synergistic activity of CPP and TRAIL in the induction of apoptosis.

Taken together, the synergistic function of combination of CPP with TRAIL in inducing apoptosis of ESCC cells were confirmed.

### CPP enhances the expression of DR4 and DR5 in ESCC cells

**Fig. 3:**
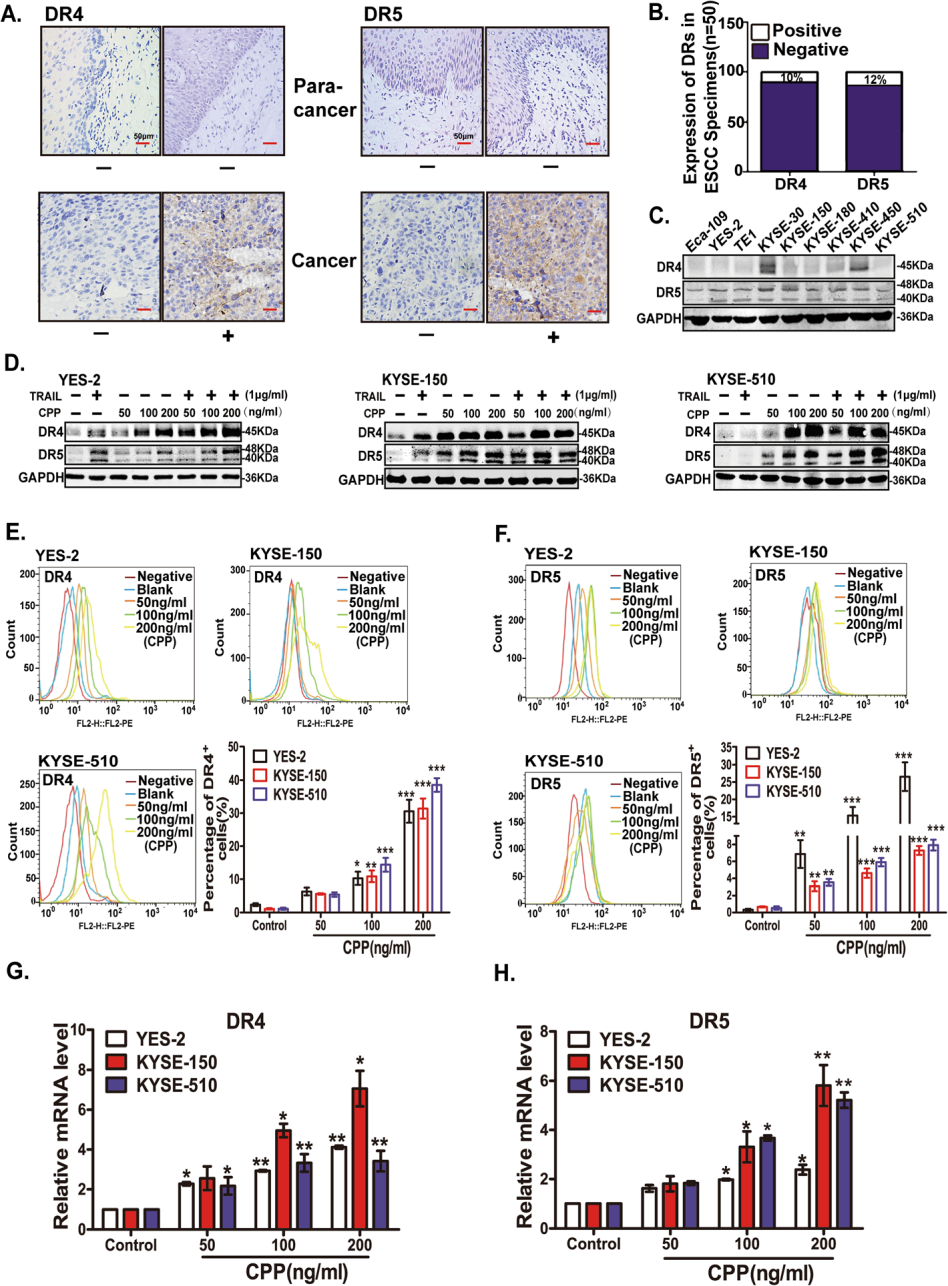
Effects of CPP on TRAIL receptors (DR4 and DR5). **a.** Immunohistochemical analysis of the expression of DR4 and DR5 in 50 cases of ESCC specimens and 33 cases of adjacent tissues of tumor. Scale bar, 50 μm. **b.** Immunohistochemical analysis of the percentage of DR4 and DR5 positive tissues in 50 cases of ESCC specimens. **c.** Western blot analysis of DR4 and DR5 protein levels in nine ESCC cells. **d.** YES-2, KYSE-150 and KYSE-510 cells were treated with various concentrations of CPP alone or in combination with TRAIL for 24 h. Changes in the expression levels of DR4 and DR5 proteins were examined by western blot. **e-f.** YES-2, KYSE-150 and KYSE-510 cells were treated with various concentrations of CPP for 24 h. Changes in the expression levels of DR4 and DR5 on the cell surface were analyzed by flow cytometry. **g-h.** YES-2, KYSE-150 and KYSE-510 cells were treated with various concentrations of CPP for 24 h. The expression of DR4 and DR5 mRNA were determined by qRT-PCR. Data are shown as the mean ± SEM. *P < 0.05, **P < 0.0.1

Binding of TRAIL to DR4 or DR5 produces a death signaling in tumor cells, and TRAIL-induced apoptosis is dependent on the expression of DRs (DR4 or DR5) at the cell membrane [9]. It has been reported that the lack of DRs is the most important reason for tumor resistance to TRAIL [9]. In order to investigate whether TRAIL resistance in esophageal cancer is also due to the deficiency of DRs, we firstly investigated the expression of DR4 and DR5 in 50 cases of ESCC specimens and 33 cases of adjacent tissues of tumor (tissues from the edge of the tumor tissue 5 cm) by immunohistochemistry. The results showed that the expression of both DR4 and DR5 in ESCC specimens and adjacent tissues is relative low (Fig. 3a and Additional file 2: Table S2). Only 10 ~ 12% of ESCC specimens were positive for DR4 or DR5 (IHC score ++ and +++) (Fig. 3b). Consistently, the expression of DR4 and DR5 was also very low in ESCC cells (Fig. 3c), accounting for the insensitivity of ESCC cell lines to TRAIL treatment.

To determine how CPP potentiates TRAIL-induced apoptosis, we investigated the effect CPP on the protein levels of DR4 and DR5. ESCC cells were treated with various concentrations of CPP (0, 50, 100 or 200 ng/ml), TRAIL (1 μg/ml), or the combination of CPP and TRAIL for 24 h. The results showed that the expression of DR4 and DR5 was significantly increased under the CPP treatment (Fig. 3d).

In addition, the expression of DR4 and DR5 on the cell membrane of ESCC cells were detected by flow cytometry. The results showed that CPP increased the expression of DR4 and DR5 in a dose-dependent manner (Fig. 3e and f).

We further evaluated how the DR4 and DR5 proteins were regulated by CPP. Firstly, DR4 and DR5 mRNA levels in ESCC cell lines were detected after treatment with different concentrations of CPP (0, 50, 100 or 200 ng/ml) for 24 h. The results showed that CPP enhanced the expression of DR4 and DR5 genes in a dose-dependent manner (Fig. 3g and h and Additional file 1: Figure S3), while the expression of decoy receptors DcR1 and DcR2 were not affected significantly (Additional file 1: Figure S4). All these results showed that CPP enhanced the transcription and expression of DR4 and DR5 significantly in ESCC cells, which contributes to the synergistic activity of CPP and TRAIL on ESCC cells.

### The CPP-induced upregulation of TRAIL receptors through downregulation of FoxP3

**Fig. 4:**
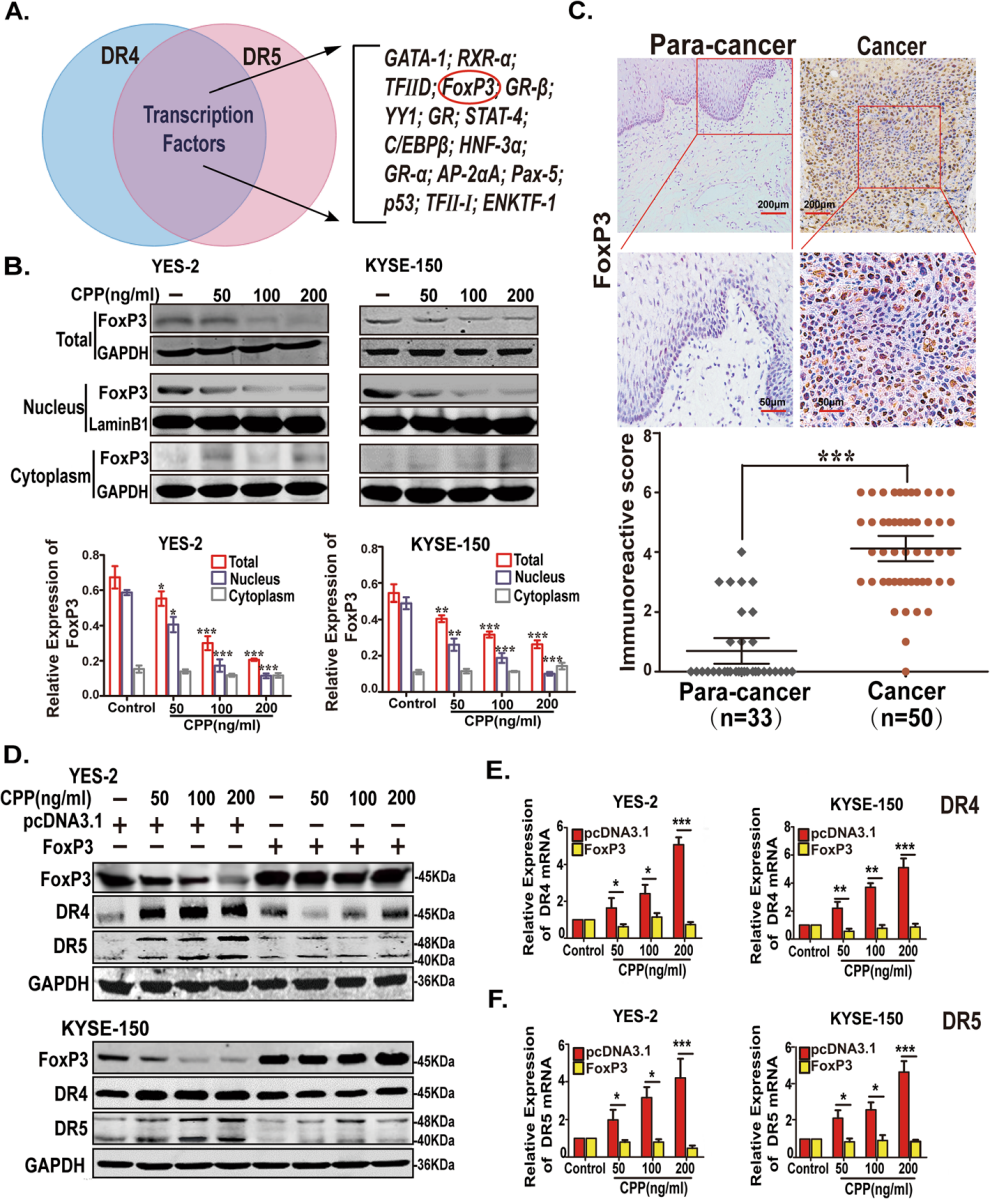
FoxP3 as the transcription repressor of DR4 and DR5. **a.** Predictions of shared transcription factors in DR4 and DR5 promoters (Version 8.3 of TRANSFAC. **b.** Western blot analysis of the change in the overall levels of FoxP3 and the change of FoxP3 in nucleus and cytoplasm after drug treatment. **c.** Immunohistochemical analysis of FoxP3 expression in 50 cases of ESCC specimens and 33 cases of adjacent specimens. Scale bar, 200 μm (above); Scale bar, 50 μm (below). **d.** Western blot analysis of the expression levels of FoxP3, DR4 and DR5 proteins. YES-2 and KYSE-150 cells were transfected with FoxP3 expression plasmid or vector (pcDNA3.1) after treatment with various concentrations of CPP alone for 24 h. **e-f.** qRT-PCR analysis of the expression levels of DR4 and DR5 mRNA. YES-2 and KYSE-150 cells were transfected with FoxP3 expression plasmid or vector (pcDNA3.1) after treatment with various concentrations of CPP alone for 24 h

Due to the elevated expression levels of DR4 and DR5 mRNA in ESCC cells after treated with CPP, we hypothesized that CPP played an essential role in upregulating DR4 and DR5 by affecting the transcription factors. Using the PROMO database (PROMO is using version 8.3 of TRANSFAC. http://alggen.lsi.upc.es/cgi-bin/promo_v3/promo/promoinit.cgi?dirDB=TF_8.3) to search binding motifs in the promoter regions of DR4 and DR5, we found that C/EBPβ, YY1 and FoxP3 were potential transcription factors shared by both DR4 and DR5 (Fig. 4a and Additional file 1:Figure S5A). To verify the effect of CPP on these three transcription factors, ESCC cells were treated with different concentrations of CPP (0, 50, 100 or 200 ng/ml), and changes in C/EBPβ, YY1 and FoxP3 mRNA levels were detected. As shown in Additional file 1:Figure S5B, FoxP3 mRNA levels was decreased significantly in a dose-dependent manner after CPP treatment, while the level of C/EBPβ and YY1 mRNA was not changed. Therefore, it is FoxP3 but not C/EBPβ or YY1 is the potential target of CPP.

We further investigate the expression level of FoxP3 protein in YES-2 and KYSE-150 cells after treatment with different concentrations of CPP (0, 50, 100 or 200 ng/ml). As a transcription factor, FoxP3 needs to translocate into the nucleus to regulate targeted genes. In order to study whether expression level and localization of FoxP3 was changed under the CPP treatment, we examined the level of Foxp3 in the nuclear, cytoplasmic and total proteins respectively. As shown in Fig. 4b, both total and nuclear FoxP3 protein levels was decreased after CPP treatment in a dose-dependent manner, while no significant change was observed in the cytoplasmic FoxP3 levels (Fig. 4b). These results suggested that CPP treatment reduced the transcriptional level of DRs by FoxP3 through inhibition of FoxP3 expression. As a result, less FoxP3 is able to translocate to the nucleus and suppress the transcription of DR4/DR5.

We further examined the expression levels of FoxP3 in ESCC specimens. FoxP3 expression was higher in ESCC specimens than that in the adjacent normal specimens (Fig. 4c, Additional file 1: Figure S6A and Additional file 2: Table S3). Furthermore, Pearson correlation analysis indicated that FoxP3 were negatively correlated with DR4/DR5 in human ESCC specimens (Additional file 2: Table S4), suggesting that higher FoxP3 level would be the reason for lower expression of DR4 and DR5 expression in ESCC tissues.

To further determine the relationship between FoxP3 and DR4/DR5 in ESCC cells, we transfected ESCC cells with a FoxP3 ectopic expression plasmid, and the FoxP3 were overexpressed successfully (Additional file 1: Figure S6B). Although CPP treatment increased the protein levels of DR4/DR5, overexpression of FoxP3 rescued CPP-induced reduction of endogenous FoxP3 and prevented the induction of DR4/DR5 transcription (Figs. 4d-f and Additional file 1: Figure S7).

Taken together, these results strongly indicated that FoxP3 negatively regulated the expression of DR4 and DR5 in ESCC cells and tissues, and acted as a transcriptional repressor of DR4 and DR5, thus downregulation of FoxP3 by CPP increased the expression of them in ESCC.

### FoxP3 bounds the promotor of DR4 and DR5 and regulated the transcriptional activity of DR4 and DR5

**Fig. 5:**
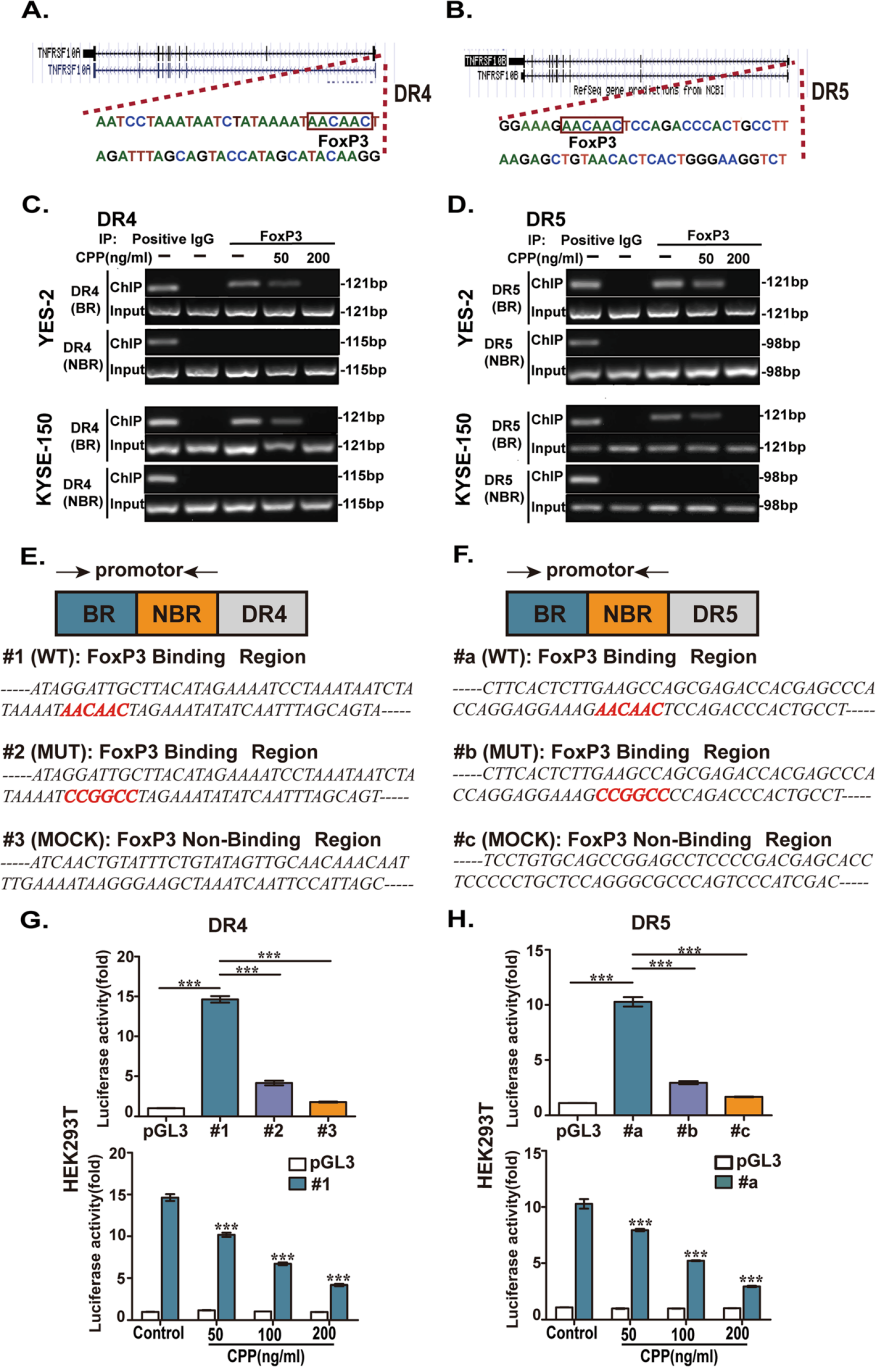
Binding of FoxP3 to the promoter region of DR4 and DR5 genes. **a-b.** Predicted binding sequence for transcription factor FoxP3 in DR4 (TNFRSF10A) and DR5 (TNFRSF10B). **c-d.** YES-2 and KYSE-150 cells were treated with various concentrations of CPP for 24 h. Then, cells were subjected to ChIP analysis with the FoxP3 antibody. The ChIP-enriched DNAs were amplified by PCR with primers specific for the binding region (BR) of FoxP3 with the DR4 or DR5 promoter. A non-binding region (NBR) was used as a negative control. **e-f.** A schematic diagram of constructed luciferase reporter genes. Luciferase reporter gene assays were conducted with contain wild FoxP3 binding site (#1 and #a), mutant FoxP3 binding site (#2 and #b) and non-containing FoxP3 binding site (#3 and #c) promoters of the DR4 and DR5 genes. **g.** Luciferase reporter assay was carried out with constructs of #1, #2, #3 in HEK293T cells and effect of CPP treatment on the activity of promoter of DR4 gene. **h.** Luciferase reporter assay was carried out with constructs of #a, #b, #c in HEK293T cells and effect of CPP treatment on the activity of promoter of DR5 gene

To confirm the binding of the FoxP3 on the promotor of DR4 and DR5, we performed ChIP assays in YES-2 and KYSE-150 cells. Firstly, using the PROMO database (PROMO is using version 8.3 of TRANSFAC. http://alggen.lsi.upc.es/cgi-bin/promo_v3/promo/promoinit.cgi?dirDB=TF_8.3), we identified the binding motif for FoxP3 in the promotor regions of DR4 and DR5 was “AACAAC” (Fig. 5a and b). As shown in Fig. 5c and d, the ChIP assay did verify that FoxP3 bound to the promotor regions of DR4 and DR5 containing the binding motif, while CPP treatment dose-dependently reduced the amount of promoter sequence associated with FoxP3. This is in consistent with the expectation of FoxP3 as a transcriptional repressor of DR4 and DR5 genes and reduction of FoxP3 levels by CPP.

We next examined whether FoxP3 directly regulates the transcriptional activity of DR4 and DR5. Luciferase reporter gene assays were conducted using DR gene promoters containing wild FoxP3 binding site or mutant FoxP3 binding site. Promoters without FoxP3 binding site were also used as controls (Fig. 5e and f). As expectation, treatment with CPP in a dose-dependent manner significantly reduced the transcriptional activity of DR4 with wild type FoxP3 binding site in the promoter (Fig. 5g). Similar results were observed for the DR5 promoter (Fig. 5h).

### Combination of TRAIL and CPP induces inhibition of the Wnt/β-catenin pathway

As described above, CPP can inhibit the expression of Survivin (Fig. 2c), which is a molecule downstream of the Wnt/β-catenin pathway [36]. We speculated that Wnt/β-catenin pathway is involved in CPP activity in esophageal cancer.

**Fig. 6:**
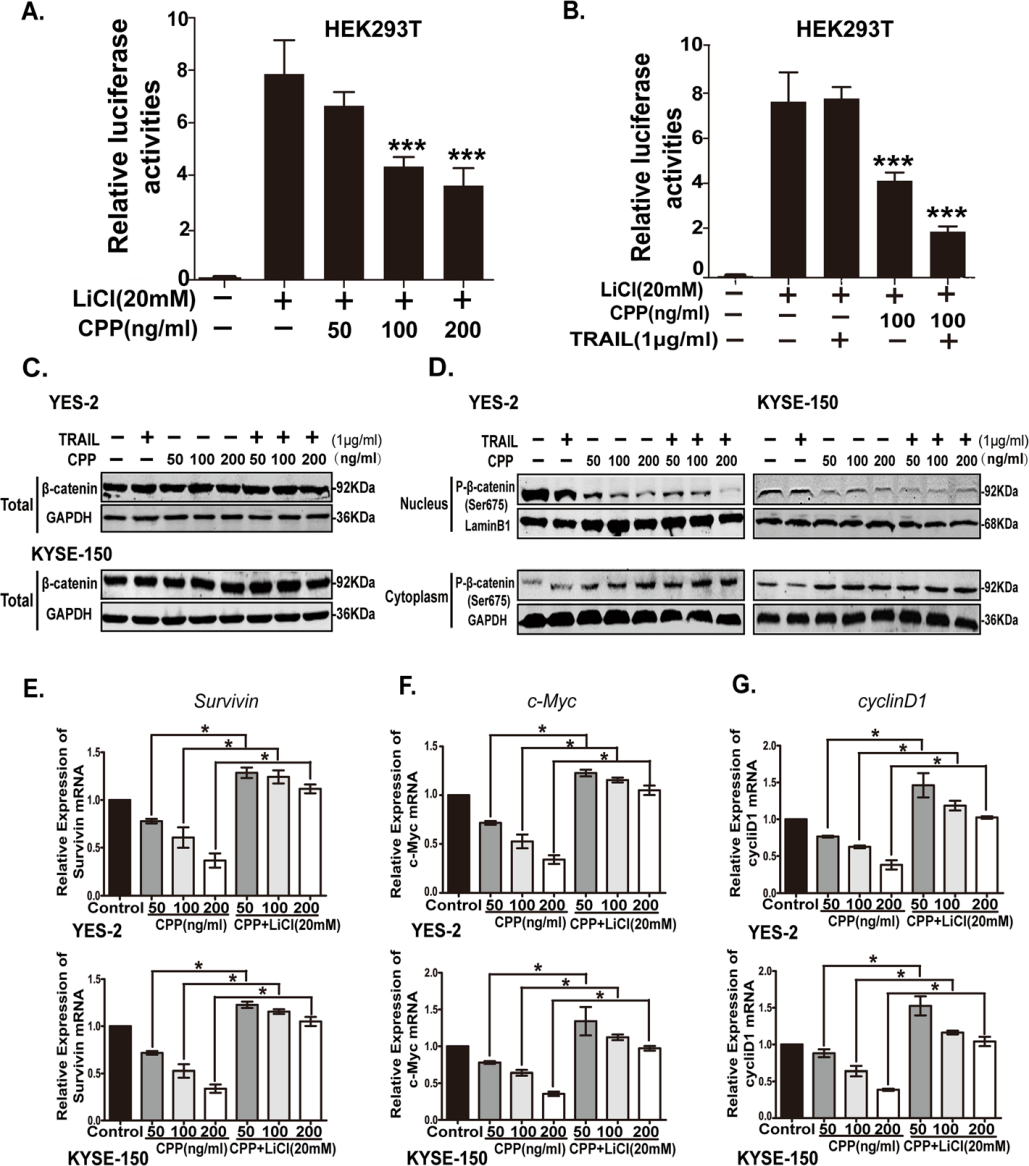
The effect of CPP on Wnt/β-catenin pathway activity. **a-b.** CPP alone (A) and combination with TRAIL (B) decreased Wnt/β-catenin activity in the TOP-flash reporter gene assay. TOP-flash contains the wild-type LEF/TCF-binding sites and can be activated by LiCl (20 mM). TOP-flash or FOP-flash (containing mutant LEF/TCF-binding sites) plasmids was co-transfected with the Renilla plasmid into cells to determine the transcriptional activity of Wnt/β-catenin signaling. **c.** Western blot analysis of the change of β-catenin in the overall levels after drug treatment of YES-2 and KYSE-150 cells. **d.** Western blot analysis of the change of p-β-catenin (Ser675) in the nucleus and cytoplasm after drug treatment of YES-2 and KYSE-150 cells. p-β-catenin (Ser675) is the form of β-catenin that enters the nucleus. **e-g.** qRT-PCR analysis of the change of Wnt/β-catenin pathway target gene (Survivin, c-Myc, and cyclin D1) mRNA levels. LiCl (20 mM) was used to activate the Wnt/β-catenin pathway after treatment of YES-2 and KYSE-150 cells with various concentrations of CPP for 24 h. Data are shown as the mean ± SEM. *P < 0.05

To confirm whether CPP could affect the activity of Wnt/β-catenin pathway in ESCC, we activated the Wnt/β-catenin pathway with LiCl (20 mM) in HEK293T cells containing the TOP/FOP-flash and Renilla plasmids, which were required for the dual-luciferase reporter assay. The results showed that CPP could inhibit the activity of Wnt/β-catenin pathway in HEK293T cells in a dose-dependent manner (Fig. 6a), while TRAIL (1 μg/ml) treatment alone had no such effect (Fig. 6b). Additionally, combined treatment with CPP and TRAIL exert the strongest inhibitory effect on the Wnt/β-catenin reporter gene activity (Fig. 6b).

To elucidate the mechanism of regulation of the Wnt/β-catenin signaling pathway by CPP and TRAIL, we further examined the expression of β-catenin, a key protein in the Wnt/β-catenin pathway. Since β-catenin can activate the expression of downstream genes including Survivin only after entering the nucleus, we examined the expression of p-β-catenin (Ser675), which represents the active form of β-catenin in the nuclear and cytoplasmic proteins respectively. Our results showed that the level of p-β-catenin (Ser675) was markedly decreased in the nucleus but increased in the cytoplasm (Fig. 6d), while no significant expression level of total β-catenin was found after CPP/TRAIL treatments (Fig. 6c). Thus, CPP treatment inhibited the activity of Wnt pathway by affecting the activation of β-catenin and its nuclear localization.

To further verify whether CPP reduces the expression of Survivin through affecting the Wnt/β-catenin pathway, the mRNA levels of Survivin, c-Myc, and cyclin D1, which are all target genes regulated by the Wnt/β-catenin pathway, were detected in the YES-2 and KYSE-150 cells in rescue experiments. Our result demonstrated that Survivin, c-Myc and cyclin D1 were all consistently decreased after treatment with CPP. However, the change was mostly abrogated once the Wnt/β-catenin pathway was activated by LiCl (20 mM) (Fig. 6e-g). These results collectively indicated that CPP treatment reduced the expression of Survivin by affecting the activity of Wnt/β-catenin pathway, thereby enhancing TRAIL-induced apoptosis.

### CPP enhances ESCC cell lines sensitivity to TRAIL in xenografted tumors in vivo

Adeno-associated virus (AAV)-based vectors are less pathogenic and thus useful as an efficient vehicle for gene delivery [34]. To further explore the effect of CPP and TRAIL in vivo, BALB/c nude mice with ESCC xenografted tumor were treated with CPP and AAV-TRAIL.

**Fig. 7:**
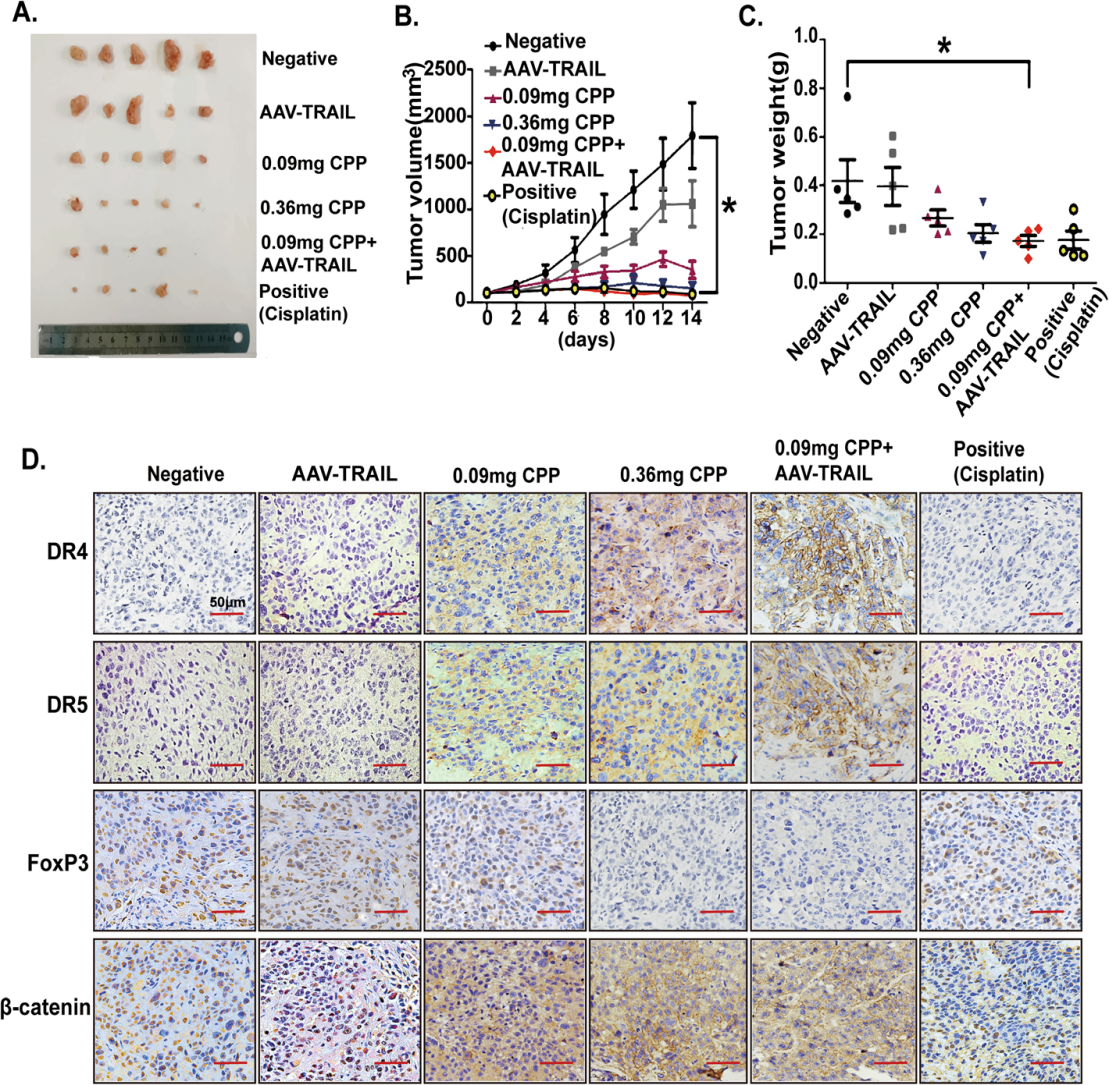
CPP increased the sensitivity of ESCC cells to TRAIL in vivo*.* KYSE-150 cells (5 × 10^6^ cells per mouse) were subcutaneously injected into 4–6 weeks BALB/c nude mice. After tumors had grown to approximately 100 mm^3^, all mice were randomly divided into six groups for drug treatment. After 14 days of treatment, the mice were sacrificed. Tumor length and width were measured with a calliper every 2 days. **a.** Images of tumors at the experimental end point. **b-c.** Growth curves of the xenograft tumors (B) and tumor weight (C) were measured at the experimental end point. *P < 0.05. **d.** The expression of DR4, DR5, FoxP3 and β-catenin in mice tumor tissues from six groups was examined by immunohistochemistry. Scale bar, 50 μm

BALB/c nude mice subcutaneously injected with KYSE-150 cells were divided into six groups. Grouping information was described in the materials and methods. As demonstrated in our study, the reduction of tumor growth was much more significant in mice treated with the combination of CPP and AAV-TRAIL than AAV-TRAIL (1 × 10^10^ Gps) or CPP (0.09 mg per mouse) alone. The anti-tumor activity of the combination treatment with CPP and AAV-TRAIL was comparable to cisplatin, a chemotherapeutic drug used clinically in ESCC treatment (Fig. 7a-c and Additional file 1: Figure S8A). Interestingly, low dose CPP plus AAV-TRAIL could achieve the similar effect on tumor growth as compared with high dose of CPP alone, suggesting that the combined application of CPP and TRAIL will allow the use of less CPP for better safety.

In addition, immunohistochemistry staining of the tumor tissues showed that the expression of DR4 and DR5 was increased, while the expression of FoxP3 was decreased significantly in CPP treatment group. Moreover, we also found that the expression of β-catenin in the nucleus was decreased while it was obviously increased in the cell cytoplasm from tumor tissues of mice treated with CPP. These results are in consistent with what we observed in vitro (Fig. 7d).

We also evaluated the potential toxic side effects of CPP alone or in combination with TRAIL through histological analysis of tissues from different organs (heart, liver, spleen, lung and kidney). No abnormal pathological morphology was observed in these tissues. This result suggests that no significant toxicity were detected in all treatment (Additional file 1: Figure S8B).

## Discussion

In the present study, we demonstrate that CPP has synergistic anti-tumor activity with TRAIL on ESCC cells through upregulating the DR4 and DR5 via a novel mechanism.

TRAIL is a promising anti-tumor drug, but it fails to demonstrate satisfactory activities in clinical trials because of its drug resistance in the treatment of many kinds of tumors. In recent years, some experiments have shown that many natural compounds could enhance the sensitivity of cancer cells to TRAIL. These drugs may upregulate the expression of DRs and FADD or downregulate anti-apoptotic proteins such as c-FLIP, XIAP or Survivin to enhance the effect of TRAIL and induce apoptosis of cancer cells [37–39]. However, there is no sufficient study about combination of TRAIL and compounds for treatment of ESCC. In this study, we found that although CPP alone demonstrates activity on tumor suppression, the combination of low concentration CPP and TRAIL achieves the same effect as high concentration CPP alone. The mechanism by which CPP mediating its effects on TRAIL-induced apoptosis appears to be associated with the upregulation of TRAIL receptors DR4 and DR5, and downregulation of Survivin, an anti-apoptotic protein [40, 41]. Furthermore, our studies firstly identified FoxP3 as an important transcription factor in the regulation DR4/5 expression. Our novel finding indicate that FoxP3 is one of transcription suppression factors of DR4/DR5, and we demonstrated that CPP-mediated the upregulation of DR4 and DR5 via inhibiting FoxP3 expression.

The potential role of FoxP3 was firstly predicted by binding motif search in the promoters of DR4 and DR5 genes. This was followed by experiment to confirm FoxP3 as a suppressor for DR4/DR5 gene transcription. Recently, an increasing number of studies have shown that FoxP3 is also expressed in cancer cells and plays vital roles in tumor progression [42]. It has been reported that FoxP3 expression is increased in thyroid cancer, hepatocellular carcinoma, colorectal cancer, cervical cancer and oral squamous cell carcinoma and promotes tumor progression [43–47]. In addition, some studies have found that FoxP3 can also reduce the sensitivity of chemotherapy drugs [48]. In this experiment, we found that FoxP3 acts as a transcriptional repressor in ESCC cells, inhibiting the expression of DR4 and DR5 at the transcriptional level. By downregulating FoxP3, CPP is able to upregulate the expression of DRs and render ESCC cells sensitive to TRAIL.

In addition to upregulating the expression of DR4/5, CPP also downregulates the expression of Survivin, which further enhances TRAIL-induced apoptosis of cells. However, CPP has minimal effect on other anti-apoptotic proteins, including c-FLIP and XIAP. Survivin is an inhibitor in the apoptosis protein family and is overexpressed in the majority of common human cancers [49–54]. As reported before, Survivin is one of the key target genes for Wnt/β-catenin pathway regulation [55]. Independently, Survivin could inhibit the activation of Caspase-3 [56]. Previously, we reported that CPP downregulated the expression of Survivin by inhibiting the activity of the Wnt/β-catenin pathway in colorectal cancer. Here, we confirmed that CPP mediated the same effect in ESCC cells. Since Caspase-3 is a downstream molecule in TRAIL-DRs signaling, our studies suggest that CPP mediated Caspase-3 activation through Wnt/β-catenin and Survivin also contributes to the enhanced TRAIL death signaling in the combination treatment group.

Canonical Wnt signaling plays crucial roles in many biological processes. A hallmark of canonical Wnt signaling is the stabilization and nuclear translocation of β-catenin [57–61]. In the absence of activation of the Wnt pathway, β-catenin in the cytoplasm is degraded by the axin complex, which consists of axin, APC, casein kinase 1 (CK1) and glycogen synthase kinase 3 (GSK3). After Wnt pathway stimulation, the Wnt ligand deactivates the axin complex to stabilize β-catenin, which is then translocated into the nucleus to form a transcriptional complex with T cell factor (TCF) to activate the expression of Wnt target genes [61], including Survivin. In the present experiment, we found that there was no significant change in the overall level of β-catenin after CPP treatment, but there appears to be an outflow of β-catenin from the nucleus to cytoplasm, which correlates with the decrease in the expression of Survivin.

**Fig. 8:**
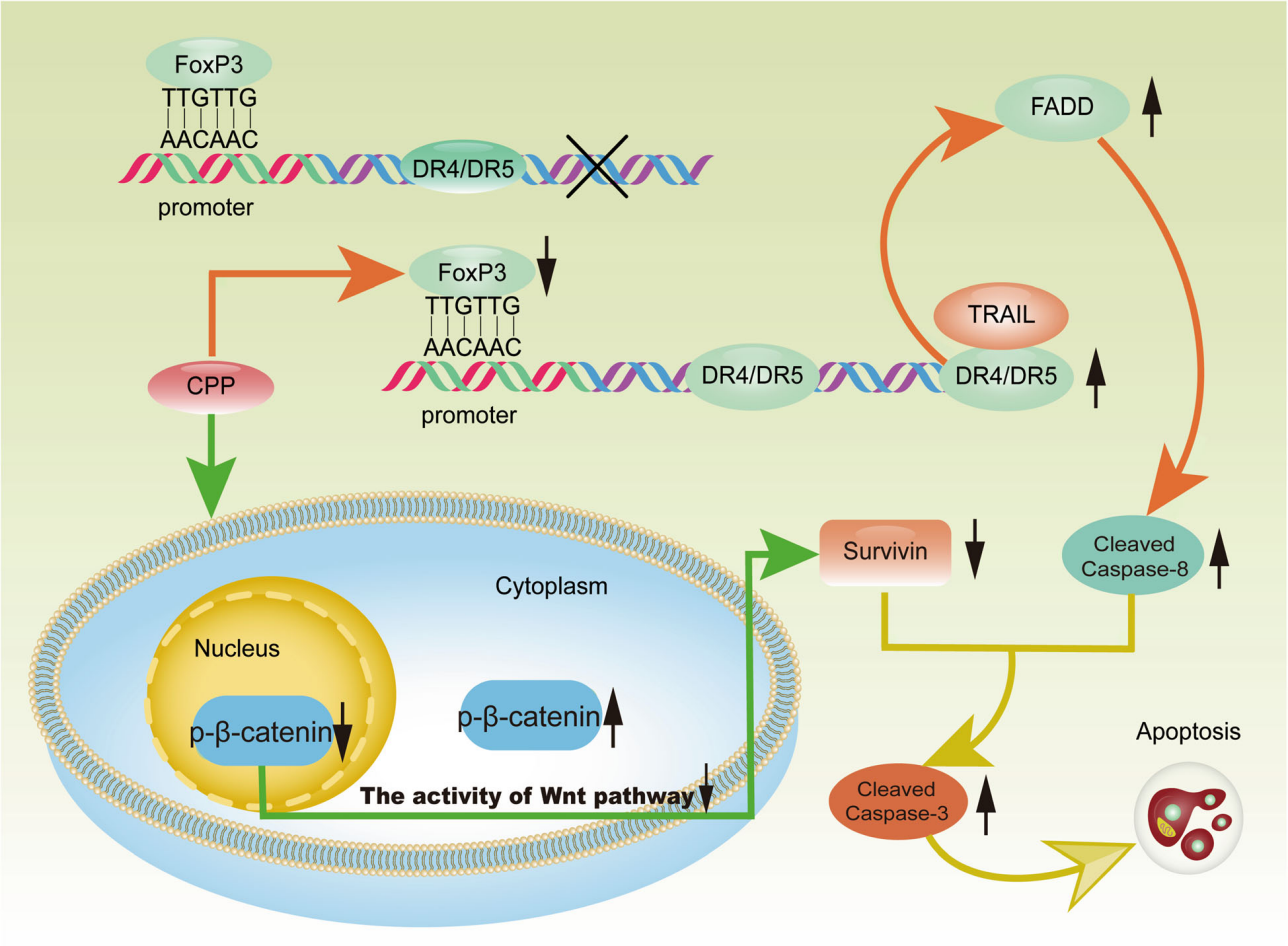
The schematic diagram depicts the mechanism of CPP-induced enhancement of sensitivity to TRAIL in ESCC cells. In ESCC cells, high expression of FoxP3 can inhibit the expression of DR4 and DR5. CPP could upregulate the expression of DR4 and DR5 through downregulating the expression of FoxP3. In the presence of a sufficient level of DR4/DR5, TRAIL could induce the downstream death signaling. On the other hand, CPP could affect the nuclear localization of β-catenin to inhibit the activity of Wnt/β-catenin pathway, thus inhibiting the transcription of Survivin, which further promotes apoptosis of ESCC cells. Therefore, CPP can enhance the sensitivity of ECSS cells to TRAIL treatment in vitro and in vivo

Taking these findings together, we conclude that CPP could upregulate DR4 and DR5 via decreasing FoxP3. In addition, CPP also reduces Survivin through downregulating the p-β-catenin in nucleus. As a result, a much powerful death signaling with TRAIL is generated, leading to high apoptosis rate of ESCC cells (Fig. 8). Considering the fact that CPP has been used as a traditional herb medicine, our studies suggest that CPP can be further investigated as a therapy for the treatment of cancer, possibly in combination with TRAIL to obtain much better efficacy for tumors that are otherwise resistant to TRAIL.

Additionally, although the mutation of TP53 is considered to be a key factor in the development of ESCC, we have not found that the expression of DR4 and DR5 was related to the mutation of TP53 in this study, neither did we investigate whether TP53 was one of targets of CPP. We might continue to evaluate this issue in our further study.

## Conclusions

In summary, our studies have identified FoxP3 as the transcriptional repressor in the regulation of DR4/5 Expression. CPP could downregulate the expression of FoxP3 in ESCC cells, thus enhance the expression of DR4 and DR5. In the presence of a sufficient level of DRs, TRAIL could induce a death signaling. On the other hand, CPP could inhibit the activity of Wnt/β-catenin pathway by affecting the nuclear localization of β-catenin in ESCC cells, thus inhibiting the transcription of Survivin, and inducing apoptosis of cancer cell. Therefore, CPP can enhance the sensitivity of ESCC cells to apoptosis induced by TRAIL in vitro and in vivo. A diagram for the mechanism of synergistic activity of CPP and TRAIL on ESCC cells is showed in Fig. 8. The above results provide a theoretical basis for the combination of CPP and TRAIL for the treatment of ESCC. Considering the safety of CPP in vivo, it is suggested that CPP should be used in combination with TRAIL, especially in tumors resistant to TRAIL.

## Supplementary information

**Additional file 1:** **Figure S1.** The effects of CPP and TRAIL alone on the viability of ESCC cells. **Figure S2.** CPP and TRAIL induces apoptosis in ESCC cells. **Figure S3.** The effects of CPP on TRAIL receptors (DR4 and DR5). **Figure S4.** The effects of CPP on TRAIL decoy receptors (DcR1 and DcR2). **Figure S5.** The transcription factors of DR4 and DR5 predicted by PROMO and the effects of CPP on selected transcription factors. **Figure S6.** The expression of FoxP3 in ESCC and adjacent tissues and the transfection efficiency of FoxP3 expression plasmid. **Figure S7.** qRT-PCR analysis of the expression levels of DR4 and DR5 mRNA. **Figure S8.** Undetectable toxicity following treatment with CPP and TRAIL in vivo.

**Additional file 2: Table S1.** The primers used for PCR **Table S2.** The expression of DR4 and DR5 in ESCC and adjacent specimens **Table S3.** The expression of FoxP3 in ESCC and adjacent specimens **Table S4.** The correlation between DR4/DR5 and FoxP3 in ESCC tissues

## Data Availability

All data generated or analyzed during this study are included in this published article**.**

## Abbreviations

AAV-TRAIL: Recombinant adeno-associated virus - soluble TRAIL
CPP: Periplocin extracted from cortex periplocae
DR4: Death receptor 4
DR5: Death receptor 5
DRs: Death receptors
ESCC: Esophageal squamous cell carcinoma
FADD: Fas-associating protein with a novel death domain
FoxP3: Forkhead box P3
TRAIL: Tumor necrosis factor-related apoptosis-inducing ligand
TUNEL: Terminal deoxynucleotidyl transferase (TdT)-mediated dUTP nick end labelingIHC Immunohistochemistry
