# Supplementary material for: Combination of the natural compound Periplocin and TRAIL induce esophageal squamous cell carcinoma apoptosis in vitro and in vivo: Implication in anticancer therapy
Source: J Exp Clin Cancer Res. 2019 Dec 21;38:501. doi: 10.1186/s13046-019-1498-z (PMC6925860; doi:10.1186/s13046-019-1498-z)
Supplement: Supplementary file 1 — Additional file 1: Figure S1. The effects of CPP and TRAIL alone on the viability of ESCC cells. Figure S2. CPP and TRAIL induces apoptosis in ESCC cells. Figure S3. The effects of CPP on TRAIL receptors (DR4 and DR5). Figure S4. The effects of CPP on TRAIL decoy receptors (DcR1 and DcR2). Figure S5. The transcription factors of DR4 and DR5 predicted by PROMO and the effects of CPP on selected transcription factors. Figure S6. The expression of FoxP3 in ESCC and adjacent tissues and the transfection efficiency of FoxP3 expression plasmid. Figure S7. qRT-PCR analysis of the expression levels of DR4 and DR5 mRNA. Figure S8. Undetectable toxicity following treatment with CPP and TRAIL in vivo. [file 13046_2019_1498_MOESM1_ESM.docx]

**Supplementary Figures**

**Supplementary Figure S1. The effects of CPP and TRAIL alone on the viability of ESCC cells. A.** ESCC cells (Eca-109, TE1, KYSE-30, KYSE-180 and KYSE-410) were treated with various concentrations of CPP and DMSO <0.01% for 24h and 48h. Cell viability was determined by MTS assay. Data are shown as the mean ± SEM. *P<0.05 compared to the untreated cell group. **B.** ESCC cells (Eca-109, TE1, KYSE-30, KYSE-180 and KYSE-450) were treated with various concentrations of TRAIL for 24h and 48h. Cell viability was determined by MTS assay. Data are shown as the mean ± SEM.

**Supplementary Figure S2.** **CPP and TRAIL induces apoptosis in ESCC cells. A.** Flow cytometry analysis of KYSE-150 and KYSE-510 cells treated with various concentrations of CPP alone and in combination with TRAIL for 24h. The cells were examined by Annexin V-PE/7-AAD double staining. The lower right and the upper right quadrants indicate the percentage of apoptotic cells. **B.** TUNEL staining of KYSE-150 and KYSE-510 cells treated with CPP (100 ng/ml), TRAIL (1 μg/ml) and CPP combination with TRAIL for 24h. The 2% DMSO treatment group served as a positive control. TUNEL-positive cells that show green fluorescence represent apoptotic cells. Scale bar, 25 μm.

**Supplementary Figure S3. The effects of CPP on TRAIL receptors (DR4 and DR5).** qRT-PCR analysis of the expression of DR4 and DR5 mRNA in ESCC cells treated with CPP. Eca-109, TE1, KYSE-30, KYSE-180 and KYSE-410 cells were treated with various concentrations of CPP for 24h. Data are shown as the mean ± SEM. *P<0.05, **P<0.01.

**Supplementary Figure S4. The effects of CPP on TRAIL decoy receptors (DcR1 and DcR2).** qRT-PCR analysis of the expression of DcR1 and DcR2 mRNA in ESCC cells treated with CPP. Eca-109, YES-2, TE1, KYSE-30, KYSE-150, KYSE-180, KYSE-410 and KYSE-510 cells were treated with various concentrations of CPP for 24h. Data are shown as the mean ± SEM.

**Supplementary Figure S5. The transcription factors of DR4 and DR5 predicted by PROMO and the** **effects of CPP on selected transcription factors. A.** Red font indicates transcription factors common to DR4 and DR5. The transcription factors from inside the red circle were selected and verified. **B.** qRT-PCR analysis of the expression of C/EBPβ, YY1 and FoxP3 mRNA in ESCC cells treated with CPP. YES-2, KYSE-150 and KYSE-510 cells were treated with various concentrations of CPP for 24h. Data are shown as the mean ± SEM. *P<0.05.

**Supplementary Figure S6. The expression of FoxP3 in ESCC and adjacent tissues and the transfection efficiency of FoxP3 expression plasmid.** **A.** Immunohistochemical analysis of the expression of FoxP3 in 50 cases of ESCC specimens and 33 cases of adjacent normal specimens. **B.** qRT-PCR analysis of the expression levels of FoxP3 mRNA. YES-2, KYSE-150 and KYSE-510 cells were transfected with the FoxP3 expression plasmid or vector (pcDNA3.1) for 24h. Data are shown as the mean ± SEM. *P<0.05.

**Supplementary Figure S7. qRT-PCR analysis of the expression levels of DR4 and DR5 mRNA.** KYSE-510 cells were transfected with FoxP3 expression plasmid or vector (pcDNA3.1) after treatment with various concentrations of CPP alone for 24h.

**Supplementary Figure S8. Undetectable toxicity following treatment with CPP and TRAIL in vivo. A.** Images of mice at the experimental end point. **B.** Morphological analysis of heart, liver, spleen, lung and kidney tissues by H&E staining. Scale bar, 50 μm.

**Figure S1**

**
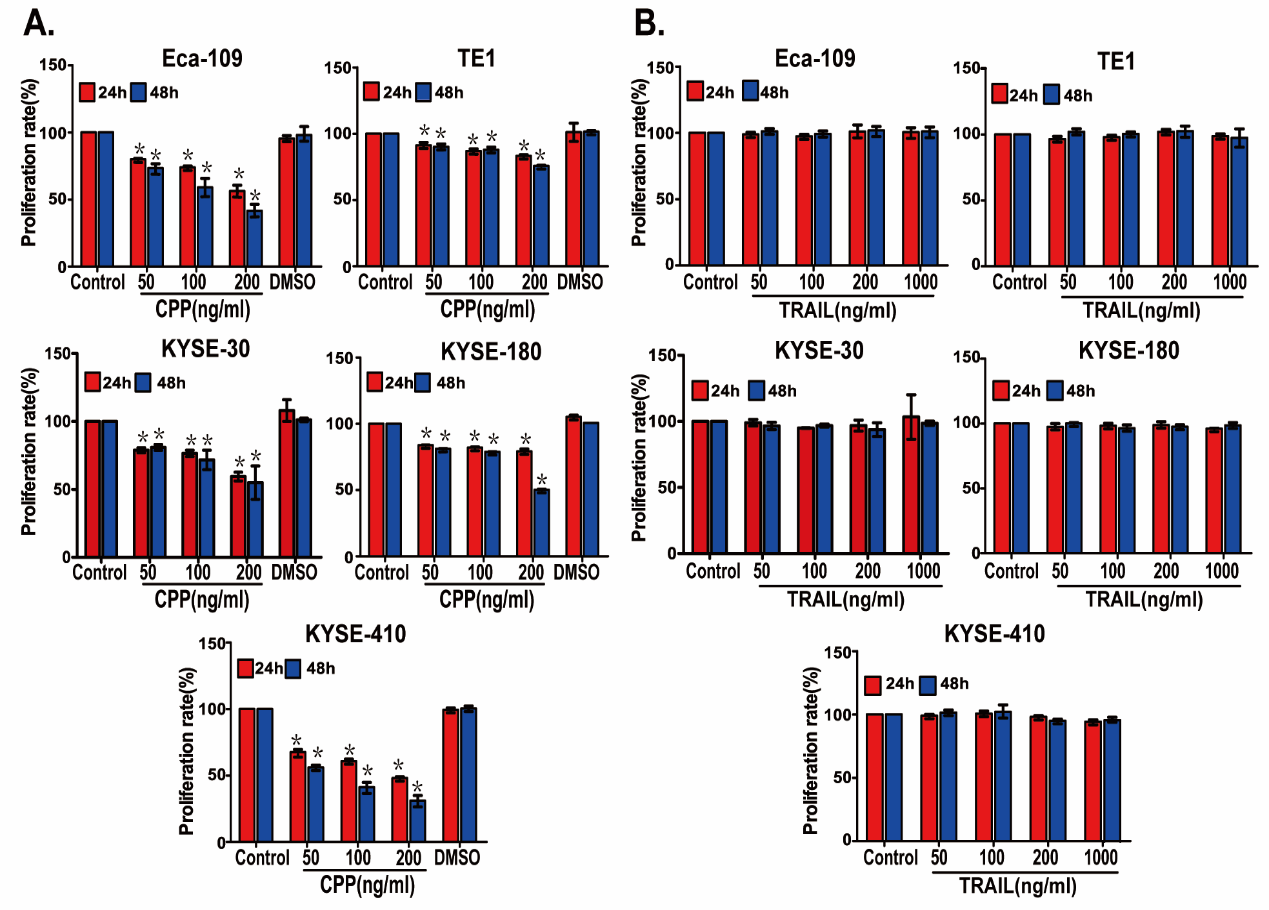
**

**Figure S2**

**­­­
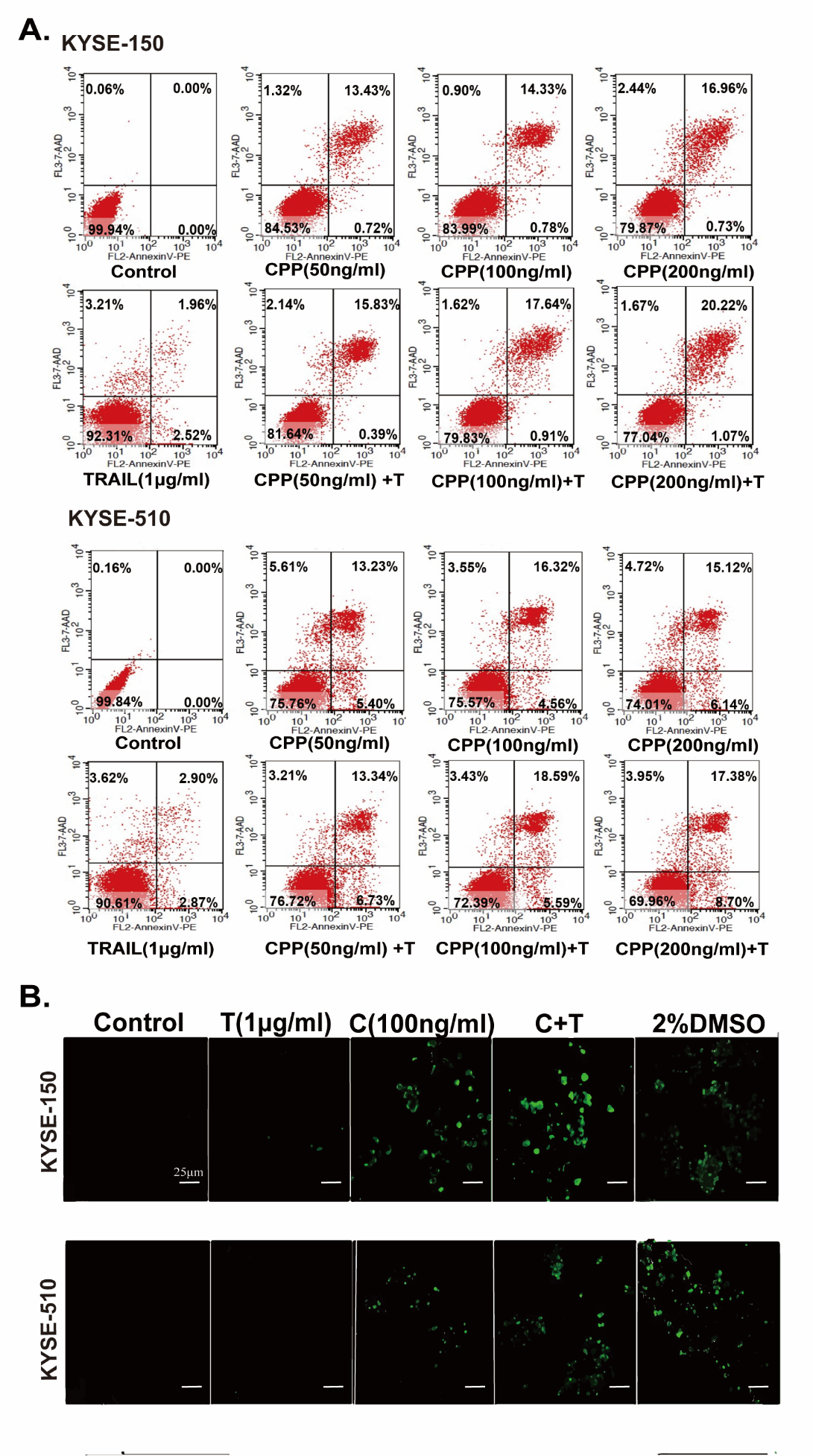
**

**Figure S3**

**
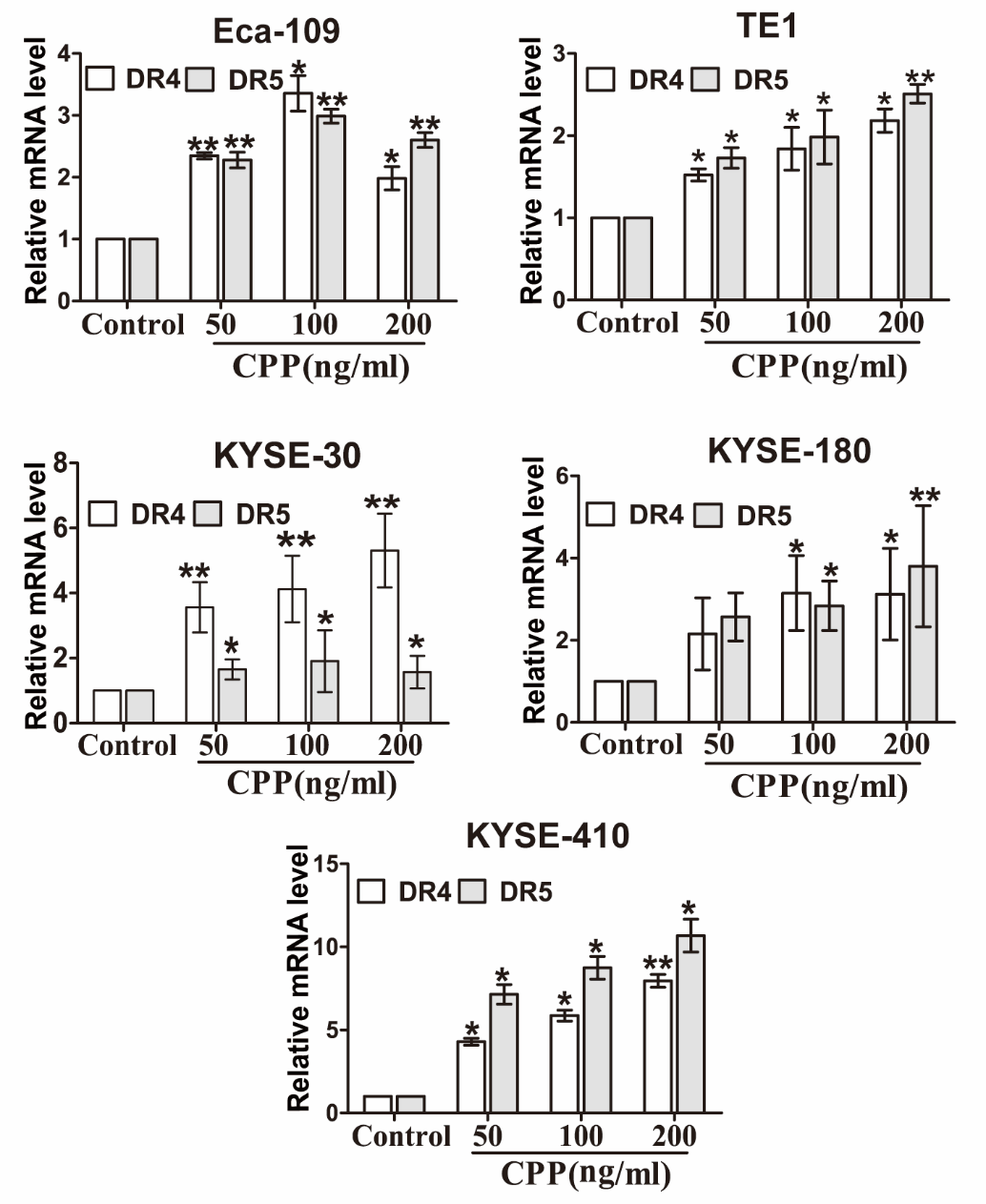
**

**Figure S4**

**
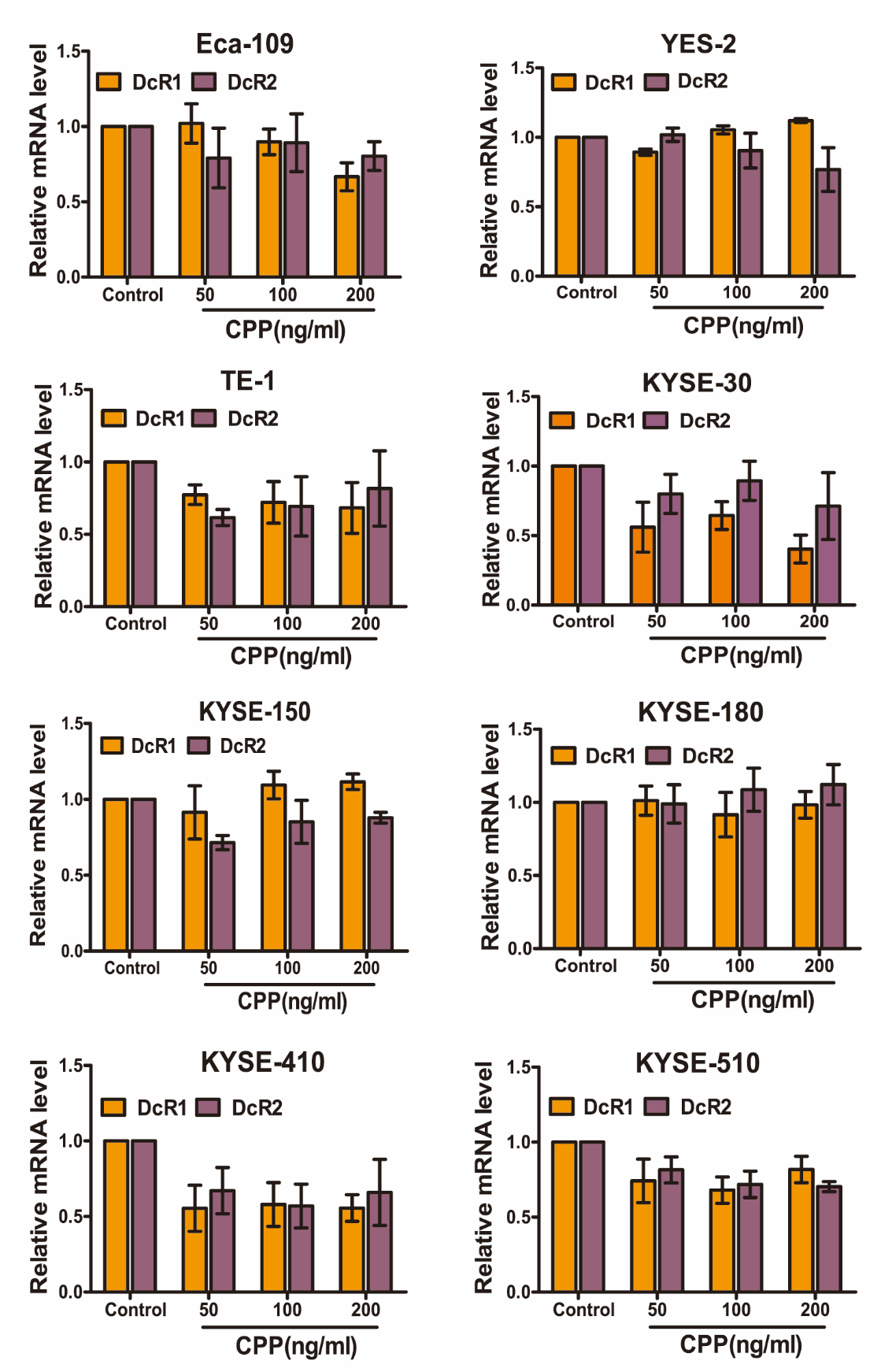
**

**Figure S5**

**
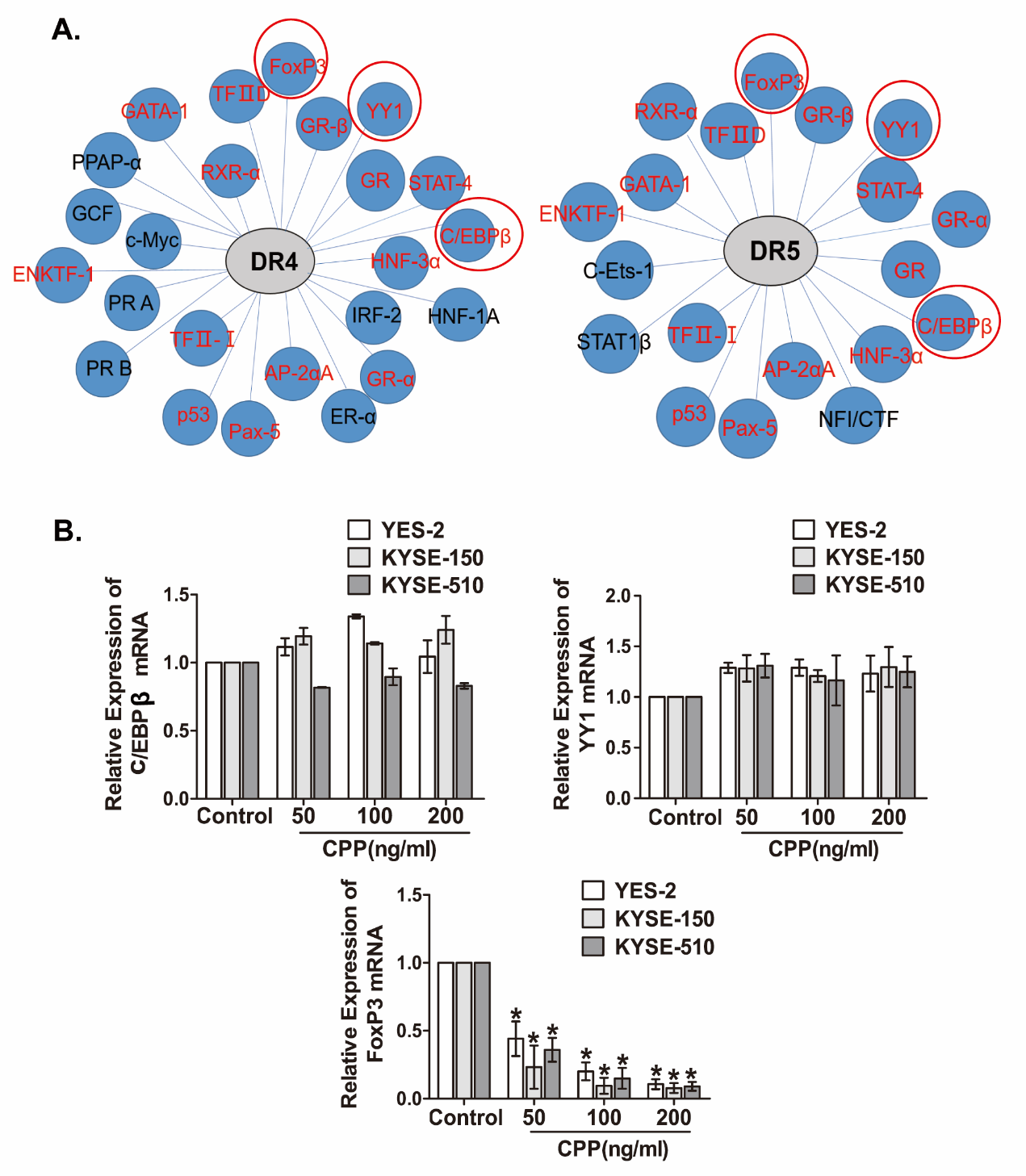
**

**Figure S6**

**
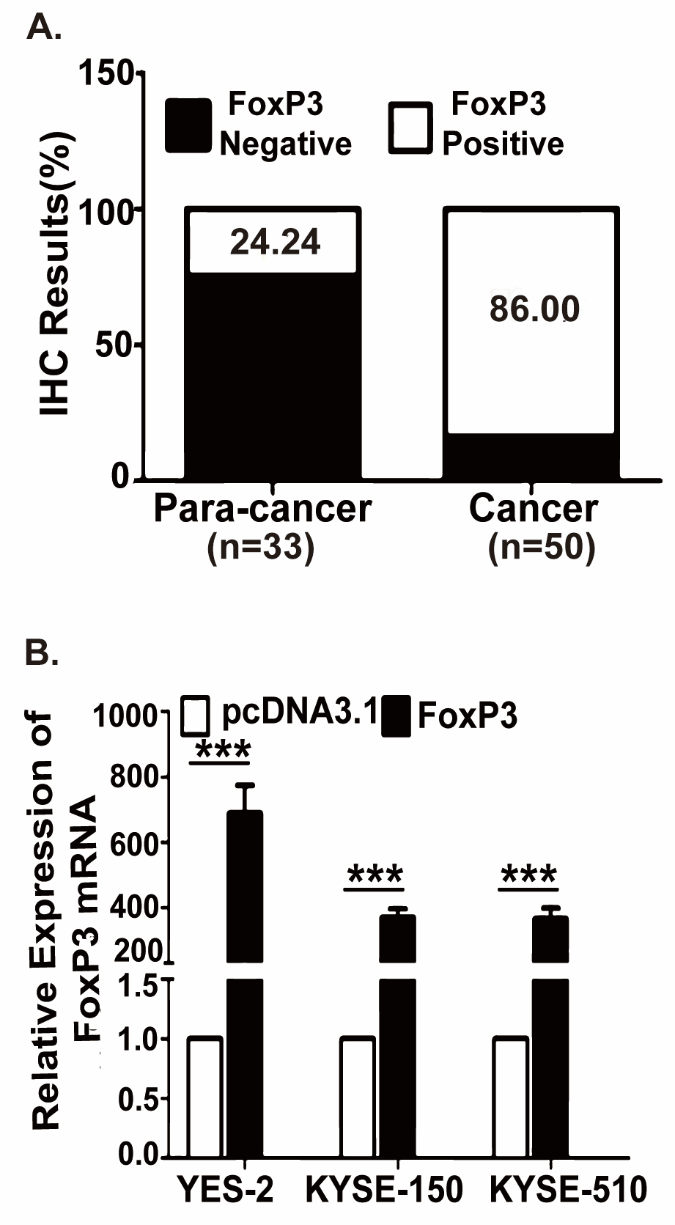
**

**Figure S7**

**
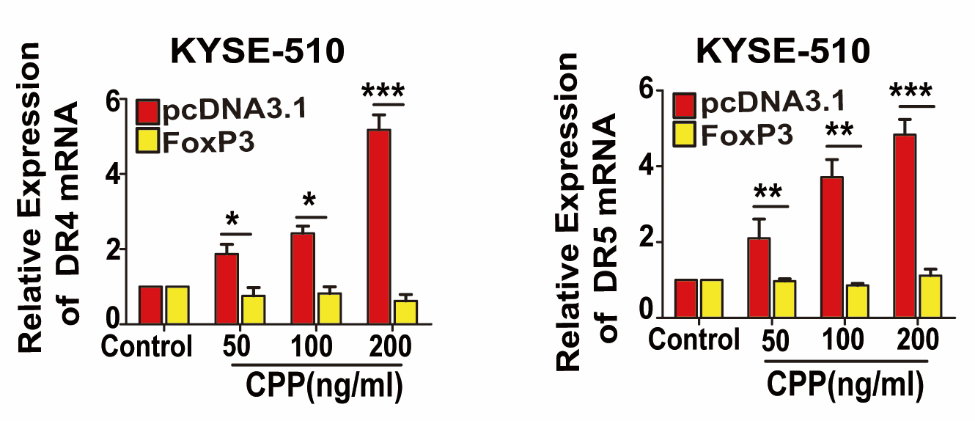
**

**Figure S8**

**
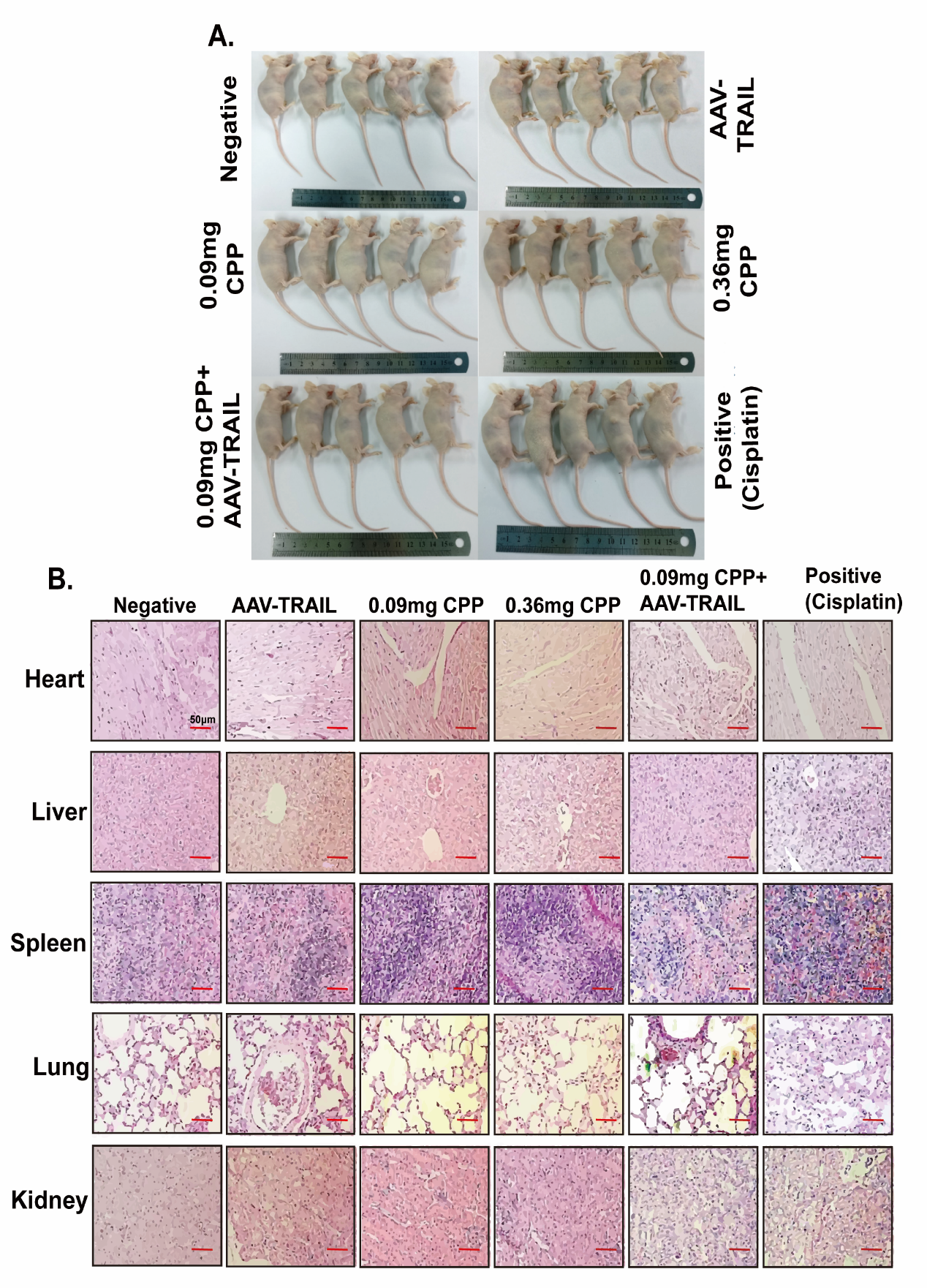
**
