# Supplementary material for: Combination of the natural compound Periplocin and TRAIL induce esophageal squamous cell carcinoma apoptosis in vitro and in vivo: Implication in anticancer therapy
Source: J Exp Clin Cancer Res. 2019 Dec 21;38:501. doi: 10.1186/s13046-019-1498-z (PMC6925860; doi:10.1186/s13046-019-1498-z)
Supplement: Supplementary file 2 — Additional file 2: Table S1. The primers used for PCR Table S2. The expression of DR4 and DR5 in ESCC and adjacent specimens Table S3. The expression of FoxP3 in ESCC and adjacent specimens Table S4. The correlation between DR4/DR5 and FoxP3 in ESCC tissues [file 13046_2019_1498_MOESM2_ESM.docx]

**Supplementary Tables**

**Supplementary Table S1: The primers used for PCR**

| **Genes** | **Primers** |
| --- | --- |
| DR4 | forward, 5’- GCAAACTTGTCAAAGAACAGCATC -3’  reverse, 5’- CTGAAGGGTCTCAGAGGAGG -3’ |
| DR5 | forward, 5’- CGGTTTTGTTGACCCACTTTA -3’  reverse, 5’- GCAGACTTGGTGCCCT-TTGAC -3’ |
| c/EBPβ | forward, 5’- TTGAACAAGTTCCGCAGGGTG -3’  reverse, 5’- AGCACAGCGACGAGT-ACAAGA -3’ |
| YY1 | forward, 5’- CACACAGAGGGAAGACCAGGC -3’  reverse, 5’- AAACCAAATAACCAACGACCACAAG -3’ |
| FoxP3 | forward, 5’- CTCCTACCCCACTGCTGGCAAAT -3’  reverse, 5’- CCCTGCCCTTCTCATCCAGA -3’ |
| Survivin  c-Myc  cyclin D1  GAPDH | forward, 5’- GTCAGAAAGCC-ACAGTTAG -3’  reverse, 5’- ACAGCCATGAGTTACGAG -3’  forward, 5’- TCCTTGCA-GCTGCTTAGACGC -3’  reverse, 5’- TGCACCGAGTCGTAGTCGAGG -3’  forward, 5’- CGCAAACACGCGCAGACCTTC -3’  reverse, 5’- TTCAGGCCTTGCACTGCG-GCC -3’  forward, 5’- GGACCTGACCTGCCGTCTAG -3’  reverse, 5’- GTAGCCCAGGAT-GCCCTTGA -3’ |

**Supplementary Table S2: The expression of DR4 and DR5 in ESCC and adjacent specimens**

| **DR4** | **Negative** | | **Positive** | | **Total** |
| --- | --- | --- | --- | --- | --- |
|  | **－** | **＋** | **＋＋** | **＋＋＋** |  |
| **Para-cancer** | **29** | **4** | **0** | **0** | **33** |
| **Cancer** | **7** | **38** | **5** | **0** | **50** |
| **Total** | **36** | **42** | **5** | **0** | **83** |

| **DR5** | **Negative** | | **Positive** | | **Total** |
| --- | --- | --- | --- | --- | --- |
|  | **－** | **＋** | **＋＋** | **＋＋＋** |  |
| **Para-cancer** | **29** | **4** | **0** | **0** | **33** |
| **Cancer** | **11** | **33** | **4** | **2** | **50** |
| **Total** | **40** | **37** | **4** | **2** | **83** |

**Supplementary Table S3: The expression of FoxP3 in ESCC and adjacent specimens**

| **FoxP3** | **Negative** | | **Positive** | | **Total** |
| --- | --- | --- | --- | --- | --- |
|  | **－** | **＋** | **＋＋** | **＋＋＋** |  |
| **Para-cancer** | **21** | **4** | **8** | **0** | **33** |
| **Cancer** | **1** | **6** | **19** | **24** | **50** |
| **Total** | **22** | **10** | **27** | **24** | **83** |

**Supplementary Table S4：The correlation between DR4/DR5 and FoxP3 in ESCC tissues**

| **DR4** | **FoxP3** | |  |
| --- | --- | --- | --- |
|  | **Negative(-/+)** | **Positive(++/+++)** | **Total** |
| **Negative(-/+)** | **3** | **42** | **45** |
| **Positive(++/+++)** | **4** | **1** | **5** |
| **Total** | **7** | **43** | **50** |

**Pearson correlation coefficient= -0.458**，P=0.007**

| **DR5** | **FoxP3** | |  |
| --- | --- | --- | --- |
|  | **Negative(-/+)** | **Positive(++/+++)** | **Total** |
| **Negative(-/+)** | **6** | **38** | **44** |
| **Positive(++/+++)** | **1** | **5** | **6** |
| **Total** | **7** | **43** | **50** |

**Pearson correlation coefficient= -0.524**，P=0.002**
